# Quality appraisal of generic self-reported instruments measuring health-related productivity changes: a systematic review

**DOI:** 10.1186/1471-2458-14-115

**Published:** 2014-02-04

**Authors:** Cindy YG Noben, Silvia MAA Evers, Frans J Nijhuis, Angelique E de Rijk

**Affiliations:** 1 CAPHRI School of Public Health and Primary Care, Department of Health Services Research, Faculty of Health, Medicine and Life Sciences, Maastricht University, Maastricht, The Netherlands; 2 Department of Work and Organizational Psychology, Faculty of Psychology and Neuroscience, Maastricht University, Maastricht, The Netherlands; 3 CAPHRI School of Public Health and Primary Care, Department of Social Medicine, Faculty of Health, Medicine and Life Sciences, Maastricht University, Maastricht, The Netherlands

**Keywords:** Absenteeism, Presenteeism, Disability, Productivity, Self-report, Psychometrics, Validation

## Abstract

**Background:**

Health impairments can result in disability and changed work productivity imposing considerable costs for the employee, employer and society as a whole. A large number of instruments exist to measure health-related productivity changes; however their methodological quality remains unclear. This systematic review critically appraised the measurement properties in generic self-reported instruments that measure health-related productivity changes to recommend appropriate instruments for use in occupational and economic health practice.

**Methods:**

PubMed, PsycINFO, Econlit and Embase were systematically searched for studies whereof: (i) instruments measured health-related productivity changes; (ii) the aim was to evaluate instrument measurement properties; (iii) instruments were generic; (iv) ratings were self-reported; (v) full-texts were available. Next, methodological quality appraisal was based on COSMIN elements: (i) internal consistency; (ii) reliability; (iii) measurement error; (iv) content validity; (v) structural validity; (vi) hypotheses testing; (vii) cross-cultural validity; (viii) criterion validity; and (ix) responsiveness. Recommendations are based on evidence syntheses.

**Results:**

This review included 25 articles assessing the reliability, validity and responsiveness of 15 different generic self-reported instruments measuring health-related productivity changes. Most studies evaluated criterion validity, none evaluated cross-cultural validity and information on measurement error is lacking. The Work Limitation Questionnaire (WLQ) was most frequently evaluated with moderate respectively strong positive evidence for content and structural validity and negative evidence for reliability, hypothesis testing and responsiveness. Less frequently evaluated, the Stanford Presenteeism Scale (SPS) showed strong positive evidence for internal consistency and structural validity, and moderate positive evidence for hypotheses testing and criterion validity. The Productivity and Disease Questionnaire (PRODISQ) yielded strong positive evidence for content validity, evidence for other properties is lacking. The other instruments resulted in mostly fair-to-poor quality ratings with limited evidence.

**Conclusions:**

Decisions based on the content of the instrument, usage purpose, target country and population, and available evidence are recommended. Until high-quality studies are in place to accurately assess the measurement properties of the currently available instruments, the WLQ and, in a Dutch context, the PRODISQ are cautiously preferred based on its strong positive evidence for content validity. Based on its strong positive evidence for internal consistency and structural validity, the SPS is cautiously recommended.

## Background

When information about the costs of alternative treatments is to be used to guide healthcare policy decision making, it is the total budget needed to treat patients with the disease that is relevant. Estimates of these total costs are based on various cost categories, such as direct health care costs (costs of healthcare resources used by patients) and indirect healthcare costs (costs due to lost productivity). As it is known, health impairments among workers can result in considerable costs due to work disability, sickness absence, and productivity loss at work, all imposing a substantial financial burden for the employee, employer and society as a whole. Various studies on different diseases have shown that indirect costs, henceforth called ‘productivity costs’, contributed substantially to total costs, illustrating how important the consequences of disease are for work performance [1-4]. Productivity costs refer to the costs associated with lost or impaired ability to work or to engage in leisure activities due to morbidity and lost economic productivity due to death [5]. A study in the Netherlands showed that the productivity costs due to low back pain can be as high as 93% of the total costs of this impairment [3]. In Germany, productivity costs due to asthma amounted to 75% of the total costs [2]. A large study in the USA among workers with common health conditions showed that productivity costs substantially exceeded the direct costs. Moreover, presenteeism costs, or costs due to reduced productivity while still at work, appeared to represent up to 60% of all costs [4].

Converting the changes of health-related productivity into a financial metric makes these changes more interpretable. However, there is no agreement on how to quantify time lost due to health impairments or how to assign a monetary value to the lost productivity. To help improve the comparability and interpretability of productivity changes, a sound estimation of productivity costs requires sound measurements of the relevant components. The comparability of estimated productivity costs is hampered by substantial differences in the costs of the items considered and the methods used for measuring sickness absence and presenteeism, as well as differences in and insufficient methodology used in the valuation of these measurement tools.

In the last decades, a large number of measurement methods and instruments have been developed to quantify health-related productivity changes. These instruments are preferably self-administered by workers with health impairments because objective measurements of productivity changes are unable to capture reduced productivity while still at work (i.e. presenteeism). Several studies have shown that presenteeism contributes substantially to the estimated total costs of health impairments among workers [6-10]. The comparability across studies estimating productivity changes and associated costs is poor, since methods of measuring changed productivity seem to vary considerably.

There is thus an urgent need for practical and applicable knowledge and insight into the reliability, validity and responsiveness of these instruments. Regarding the validity of the instruments, one should keep in mind that the extent to which a valid measurement of productivity loss, especially presenteeism, can be achieved is often influenced by many factors (e.g. the amount of teamwork required in the job, the work setting, the desired actual production output, etc.) [6,11].

Although several researchers have provided comprehensive reviews of existing instruments that measure productivity changes, the methodological quality of the reviewed studies remains unclear [12,13]. Consequently, judgements on the quality of the studies cannot be made. If the methodological quality of a study on the measurement properties of a specific instrument is appropriate, the results can be used to assess the quality of the instrument at issue, including its measurement properties. However, if the methodological quality of the study is inadequate, the results cannot be trusted and the quality of the instrument under study remains unclear, despite of the magnitude or strength of the estimates presented [14]. Therefore, in this systematic review both the methodological quality of the study and the quality of the instrument, based on its psychometric properties, are taken into account. The main aim of this systematic review is therefore to critically appraise and compare the measurement properties of generic, self-reported instruments measuring health-related productivity changes.

## Methods

### Search strategy

A systematic review was conducted of studies evaluating the measurement properties of generic, self-reported health-related productivity instruments. In February 2013, the following relevant electronic databases were searched for English-language peer-reviewed journal articles: Medline (PubMed), PsychINFO (EBSCOhost), Embase and EconLit. The search query (see Additional file 1: Table S1) consisted of a combination of related terms for the following features: the construct of interest (i.e. workplace productivity loss OR absenteeism OR presenteeism) ‘AND’ studies on measurement properties ‘AND’ the instrument of interest (i.e. generic AND self-report). The complete search strategies, including a sensitive search filter and exclusion filter [15] can be obtained via the corresponding author. No restrictions for the year of publication were made. Additional relevant studies were identified by performing database searches using the names of retrieved instruments and information from the retrieved reference lists.

### Eligibility criteria

Study selection was based on the following eligibility criteria: (i) the aim of the study is the development or evaluation of the measurement properties of the instrument; (ii) the instrument under review measures health-related productivity changes; (iii) the instrument is generic and thus not solely focus on productivity changes due to a specific health impairment; (iv) health-related productivity change is rated from a worker’s perspective (i.e. is self-reported); (v) full-text articles published in English were available.

### Selection process

Three reviewers (CYGN, SE and AER) independently determined the eligibility of all studies based on the title, keywords and abstracts. Studies in which an instrument of potential interest was used as an outcome measure, such as in intervention trials, were excluded. Review articles were excluded as well.

All reference lists and instruments of interest mentioned in all articles (both included and excluded from the review) were used in a secondary database search to identify additional relevant studies. If there was any doubt as to whether the studies met the eligibility criteria after individual selection, discrepancies were resolved through discussion between the reviewers, and a consensus decision was made. In case of remaining uncertainty, the full text was reviewed. Reasons for excluding the abstracts can be retrieved from the first author.

### Measurement properties

The Consensus-based Standards for the selection of health Measurement Instruments (COSMIN) covers relevant measurement properties for health-related, patient-reported outcomes and is based on international consensus [14,16,17].

The COSMIN checklist was used to rate the methodological quality of each article evaluating measurement properties. It covers three main quality domains: reliability, validity and responsiveness. Each domain is divided into different measurement properties and aspects with questions ranking the quality level of the design and statistical analyses [16]. Table 1 illustrates the most appropriate measures to critically appraise the measurement properties.

**Table 1:** Description of the measurement domains, properties, aspects, and statistics and methods

**Domains**	**Properties**	**Aspects**	**Statistics/Methods**
Reliability			
	Internal consistency		Cronbach’s alpha or Kuder-Richardson formula (KR-20) to determine relevance
			Factor analysis or principal component analysis to determine whether items form one or more than one scale
	Reliability		Intraclass correlation coefficient (ICC) or Cohen’s kappa
	Measurement error		Standard error of measurement (SEM)
			Smallest detectable change (SDC)
			Change beyond measurement error
			Limits of agreement (LoA)
			Minimal important change to determine the adequacy of measurement error
Validity			
	Content validity	Face validity	Assessment of relevance of all items for the construct, aim and target group
			Assessment of important missing items
	Construct validity		
		Structural validity	Factor analysis to confirm the number of subscales present
		Hypotheses testing	Assessment of a priori hypotheses, clearly indicating both direction and magnitude of the correlation or difference
		Cross-cultural validity	Assessment of adequate reflection of the performance of the items of the original instrument
	Criterion validity		Correlation
			Area under the receiver operator characteristics curve (AUC)
			Sensitivity and specificity
Responsiveness			
			Assessment of a priori hypotheses focussing on the change score of an instrument in the hypotheses
			Area under the receiver operator characteristic curve (AUC)

The domain ‘reliability’ is defined as the degree to which the measurement is free from error and the degree to which patient scores have not changed for repeated measurements over different sets of items from the same questionnaire (internal consistency), over time (test-retest), over different persons (inter-rater), or over different occasions (intra-rater). Reliability is further assessed based on the measurement properties: (i) internal consistency: the degree of interrelatedness among the items: (ii) reliability: the proportion of total variance in the measurement due to ‘true’ (free of error) differences between patients; and (iii) measurement error: systematic and random error of patient scores (not attributed to true changes in the construct to be measured).

The second domain, ‘validity’, is described as the degree to which the instrument measures what it purports to measure. Three measurement properties are assessed: (i) content validity: the degree to which content of the instrument adequately reflects the construct to be measured, including face validity; (ii) construct validity, or the degree to which the scores of the instrument are consistent with the hypotheses, is divided into three aspects: (a) structural validity: the degree to which the scores of the instrument are an adequate reflection of the dimensionality of the construct to be measured; (b) hypotheses testing: the degree to which scores of the instrument are consistent with hypotheses; (c) cross-cultural validity: the degree to which performance of the items on a translated or culturally adapted instrument are an adequate reflection of the performance of the items of the original version of the instrument; and finally (iii) criterion validity: the degree to which the scores of the instrument are an adequate reflection of a ‘gold standard’ [17].

The third domain, ‘responsiveness’, is defined as the ability of the instrument to detect change over time in the construct to be measured.

### Data extraction

Three reviewers (CYGN, AER, SMAAE) independently extracted the target data from the full text articles. The studies (n = 25) are described based on information retrieved from the original publications and contain information regarding the study country, population, sampling methods, setting, age, gender and response rates. The instruments (n = 15) are described based on information retrieved from the original publications (e.g. content, number of items, rating, item example, recall, and discipline).

### Methodological quality assessment

To determine the methodological quality of the studies, each was assessed independently by three reviewers (CYGN, AER, SMAAE). Consensus was reached by pairing the reviewers’ results. The pairing was CYGN and SMAAE (pair 1) and CYGN and AER (pair 2). When a pair of reviewers disagreed, consensus was reached through discussion within the project group (CYGN, SMAAE, FJN, AER). The methodological quality assessment of the studies was conducted by scoring each of the nine measurement properties as presented in nine boxes by the COSMIN-checklist. A four-point rating scale was used (i.e. poor, fair, good, or excellent) to calculate the quality score per measurement property. The scores took into account, for example, the used sample size which may differ between methods (rules of thumbs for factor analyses vary between a subject-to-variables ratio of 4:1 to 10:1 or a sample size of 50 is needed to obtain confidence intervals from 0.70-0.90 around an ICC to assess reliability estimates); the missing items and the missing responses per items and how they were handled; the description of the comparator; and the appropriateness of the statistics (e.g. the internal consistency statistic only gets an interpretable meaning when the interrelatedness among the items is determined of a set of items that together form a reflective model). The methodological quality was determined per study for each measurement property separately by taking the lowest rating of the items to that measurement property in each box (worse score counts) [14].

### Best evidence synthesis

For each instrument, a best evidence synthesis was performed by combining the methodological quality score for each measurement property per instrument as assessed in this study (excellent, good, fair or poor) with the consistency of their results concerning the measurement property (positive or negative evidence for a measurement property). The ratings correspond to the Cochrane Back Review Group levels of evidence [18]. The levels of evidence are strong, moderate, limited, conflicting, or unknown. A strong level of evidence represents consistent findings in multiple studies of good methodological quality *or* in one study of excellent methodological quality; moderate levels of evidence represent consistent findings in multiple studies of fair methodological quality *or* in one study of good methodological quality; a limited level of evidence occurs when one study of fair methodological quality is being presented; an unknown level of evidence is noted when only studies of poor methodological quality are obtained and a conflicting level of evidence represents for multiple studies with conflicting findings.

## Results

### Study selection

**Figure 1:**
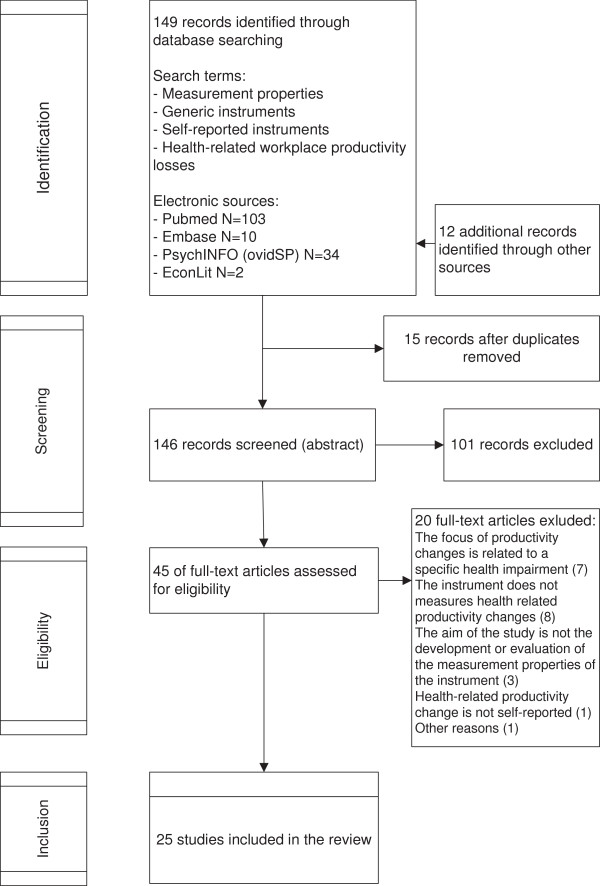
Flowchart of inclusion.

The first search in MEDLINE (PubMed) resulted in 103 hits. The searches in PsychINFO, Embase and EconLit resulted in 34, 10 and 2 hits, respectively. Manual searches based on reference lists and names of original instruments identified 12 additional records. Duplicates (N = 15) were automatically discarded via Endnote X4 resulting in 146 records to be screened. After screening abstracts and titles, 45 articles were reviewed, and 25 met the eligibility criteria. The most common reasons for exclusion were: (i) the main aim of the study was not about assessing the psychometric properties of a health-related productivity instrument; (ii) the focus of productivity changes was related to specific health impairment; (iii) the instrument did not measure health-related productivity changes; (iv) health-related productivity was not self-reported. A flow chart illustrates the process of inclusion (see Figure 1).

### General description of the identified studies

Finally, 25 articles evaluating 15 different instruments were included in this study. The characteristics of the 25 included studies are presented in Table 2. Several studies evaluated the measurement properties of multiple instruments and are therefore mentioned several times [19-21]. Two studies were conducted in the UK and Ireland [22,23], three in the Netherlands [20,24,25], three in Canada [26-28], and 17 in the USA [19,21,29-43]. The largest proportion (44%) of the studies focused on physical disorders [20,22,23,25-28,30,35,37,42]. Mental health problems were only addressed in two studies [19,29]. Populations with varying health problems (physical and mental constraints) were included in four studies [24,31,36,41]. Finally, workers with non-specified health issues [32,34,38,40,43] or workers with job-related injuries [21,33,39] were also included in five and three studies respectively. Twelve studies were conducted at the workplace [20,21,24,31-34,38-41,43] and nine studies were conducted in a healthcare institution [19,26-29,35-37,39]. One study was conducted at the workplace and at a healthcare institution and is therefore mentioned twice [39]. One study took place at an occupational medicine college [30], and four studies derived their participants from a database [22,23,25,42].

**Table 2:** Characteristics of the identified studies

**Study**	**Country**	**Instrument**	**Population**	**Sampling**	**Setting**	**Mean age (SD)**	**Gender (% female)**	**Response rate (%)**
Beaton [26]	Canada	WLQ	Musculoskeletal disorders	Convenience	Health care	42.5 (10.1)	54%	33%
Endicott [29]	USA	EWPS	Major depressive disorders	Convenience	Health care	41 (9.6)	70%	N.A.
Erickson [19]	USA	WLQ WPSI EWPS WPAI	Anxiety disorders (i) minimal-to-mild (ii) moderate-to-severe	Convenience	Health care	(i) 37.5 (12.2) (ii) 34.2 (9.8)	(i) 48.8% (ii) 75%	51%
Forst [30]	USA	AMA-guide	Low back injuries	Convenience	Academy	N.A.	N.A.	100%
Goetzel [31]	USA	WPSI	Allergies, respiratory infections, arthritis*	N.A.	Work-place	47 (N.A.)	30%	N.A.
Kessler [33]	USA	WHO HPQ	Job-related accidents-injuries (i) airline reservation agents (ii) customer service reps. (iii) executives (iv) railroad engineers	Convenience	Work-place	(i) 30-44 (2.4) (ii) 30-44 (2.2) (iii) 45-59 (2.0) (iv) 45-59 (1.7)	(i) 80.3% (ii) 47.2% (iii) 19.3% (iv) 2.4%	(i) 39% (ii) 29% (iii) 50% (iv) 57%
Kessler [32]	USA	WHO HPQ	Non-specified health status	Convenience	Work-place	N.A.	N.A.	N.A.
Koopman [34]	USA	SPS	Non-specified health status	Convenience	Work-place	46.5 (9.4)	47.9%	74%
Koopmanschap [24]	NL	PRODISQ	Musculoskeletal complaints, stress factors, back complaints**	Convenience	Work-place	N.A.	N.A.	N.A.
Kumar [35]	USA	HRPQ-D	Infectious Mononucleosis	Convenience	Health care	19.3 (3.6)	41.9%	97%
Lerner [36]	USA	WLQ	RA, chronic daily headache syndrome and epilepsy	Convenience	Health care	41.3 (11.1)	72.7%	N.A.
Lerner [37]	USA	WLQ	Osteoarthritis	Convenience	Health care	53.7 (7.1)	65.4%	30%
Meerding [20]	NL	HLQ	Musculoskeletal complaints	Convenience	Work-place	(i) 35 (N.A.)	(i) 0%	(i) 69%
		Q&Q	(i) industrial workers (ii) construction workers			(ii) 42 (N.A.)	(ii) 6%	(ii) 85%
Ozminkanski [21]	USA	WLQ WPSI	Job-related accidents-injuries	Consecutive	Work-place	37.77 (N.A.)	34.31%	48%
Prochaska [38]	USA	WBA-P	Non-specified health status	Convenience	Work-place	47.6 (11.4)	56.8%	N.A.
Reilly [39]	USA	WPAI	Job-related accidents-injuries (i) Group 1 (ii) Group 2	Convenience	Health care/Work-place	(i) 37.2 (9.7) (ii) 39.4 (11.8)	(i) 65.5% (ii) 60.8%	48%
Roy [28]	Canada	WLQ	Chronic upper-extremity disorders	Convenience	Health care	46 (9)	53%	83%
Shikiar [40]	USA	HWQ	Non-specified health status	Convenience	Work-place	N.A.	N.A.	45%
Stewart [43]	USA	WHI	Non-specified health status	Convenience	Work-place	N.A.	66%	72%
Tang [27]	Canada	WLQ	Shoulder and elbow disorders	Convenience	Health care	43.3 (11.5)	42.5%	87%
Turpin [41]	USA	SPS	Allergies, arthritis or joint pain/stiffness, asthma***	Convenience	Work-place	43.2 (N.A.)	29.4%	63%
Van Rooijen [25]	NL	HLQ	(i) Migraine (ii) spinal cord injury (iii) knee injury (iv) hip injury	Convenience	Data base	N.A.	N.A.	(i) 58% (ii) 81% (iii) 76% (iv) 75%
Walker [42]	USA	WLQ	Rheumatoid arthritis	Continuously	NDB	53.5 (10.5)	77%	98%
Zhang [22]	UK & Ireland	WPAI	Rheumatoid arthritis	Convenience	ERAN	52.1 (10)	72%	53%
Zhang [23]	UK & Ireland	VOLP	Rheumatoid arthritis	Convenience	ERAN	52 (10)	72%	81%

*WLQ* = *Work Limitations Questionnaire*; *EWPS* = *Endicott Work Productivity Scale*; *N.A*. = *Not Available*; *WPSI* = *Work Productivity Short Inventory*; *WPAI* = *Work Productivity and Activity Impairment Instrument*; *AMA*-*guide* = *American Medical Association*-*Guides*; *WHO HPQ* = *World Health Organization Health and work Performance Questionnaire*; *SPS* = *Stanford Presenteeism Scale*; *PRODISQ* = *PROductivity and Disease Questionnaire*; *HRPQ*-*D* = *Health*-*Related Productivity Questionnaire Diary*; *HLQ* = *Health and Labor Questionnaire*; *Q&Q* = *Quality and Quantity questionnaire*; *WBA*-*P* = *Well*-*Being Assessment for Productivity*; *HWQ* = *Health and Work Questionnaire*; *WHI* = *Work and Health Interview*; *VOLP* = *Valuation of Lost Productivity questionnaire*; *NDB* = *National Databank for Rheumatic Diseases* ; *ERAN* = *The Early Rheumatoid Arthritis Network*.

*Continued: asthma, anxiety disorders, depression, bipolar disorder, stress, diabetes, hypertension, migraine, other major headaches, coronary heart disease/high cholesterol.

**Continued: dyspepsia, chronic fatigue syndrome, psoriasis, rheumatoid arthritis.

***Continued: back or neck disorder, breathing disorder, depression, anxiety or emotional disorder, diabetes, hearth or circulatory problem, migraine/chronic headaches, stomach or bowel disorder.

Fifteen different generic self-reported instruments measuring health-related productivity changes are included in this study. The general characteristics of the instruments are presented below. An additional table shows a more detailed description of the instruments (see Additional file 2: Table S2).

In Table 3, the methodological quality of each study per measurement property and instrument is presented. The Work Limitations Questionnaire (WLQ) is the most frequently evaluated instrument [19,21,26-28,36,37,42], followed by the Work Productivity and Activity Impairment Instrument (WPAI) [19,22,39], and the Work Productivity Short Inventory (WPSI) [19,21,31]. Criterion validity was evaluated in 21 studies [19-23,25,28-41,43]. Hypotheses testing was evaluated in 15 studies [19,22,26-36,39,41]. Only one study addressed measurement error [39], and none of the studies evaluated cross-cultural validity.

**Table 3:** Methodological quality of each study per measurement property per instrument

**Instrument and paper**	**Internal consistency**	**Reliability**	**Measurement error**	**Content validity**	**Structural validity**	**Hypotheses testing**	**Cross-cultural validity**	**Criterion validity**	**Responsiveness**
**WLQ**									
Beaton [26]	Poor	.	.	.	.	Fair	.	.	Fair
Erickson [19]	Poor	.	.	.	.	Poor	.	Poor	Poor
Lerner [36]	Poor	Fair	.	Good	.	Poor	.	Poor	.
Lerner [37]	Good	.	.	Fair	Good	.	.	Poor	.
Ozminkowski [21]	.	.	.	.	.	.	.	Fair	.
Roy [28]	.	.	.	.	.	Fair	.	Good	Fair
Tang [27]	Fair	.	.	Fair	.	Fair	.	.	.
Walker [42]	Excellent	.	.	.	Good	.	.	.	.
**EWPS**									
Endicott [29]	Poor	Poor	.	.	.	Fair	.	Poor	.
Erickson [19]	Poor	.	.	.	.	Poor	.	Poor	Poor
**WPAI**									
Erickson [19]	Poor	.	.	.	.	Poor	.	Poor	Poor
Reilly [39]	.	Poor	Poor	.	.	Fair	.	Fair	.
Zhang [22]	.	.	.	.	.	Fair	.	Fair	.
**WPSI**									
Erickson [19]	Poor	.	.	.	.	Poor	.	Poor	Poor
Goetzel [31]	.	Poor	.	Poor	.	Poor	.	Poor	Poor
Ozminkowski [21]	.	.	.	.	.	.	.	Fair	.
**AMA**-**guides**									
Forst [30]	.	Poor	.	.	.	Poor	.	Poor	.
**WHO**-**HPQ**									
Kessler [33]	.	.	.	Good	.	Poor	.	Good	.
Kessler [32]	.	Fair	.	Good	Fair	Fair	.	Fair	Poor
**SPS**									
Koopman [34]	Good	.	.	Fair	Good	Fair	.	Fair	.
Turpin [41]	Good	Fair	.	.	Good	Fair	.	Fair	.
**PRODISQ**									
Koopmanschap [24]	.	.	.	Excellent	.	.	.	.	.
**HRPQ**-**D**									
Kumar [35]	.	Fair	.	Poor	.	Poor	.	Fair	Fair
**HLQ**									
Meerding [20]	.	Fair	.	.	.	.	.	Fair	.
van Roijen [25]	.	.	.	.	.	.	.	Fair	.
**Q&Q**									
Meerding [20]	.	Fair	.	.	.	.	.	Fair	.
**WBA**-**P**									
Prochaska [38]	.	.	.	.	Good	.	.	Good	.
**HWQ**									
Shikiar [40]	Fair	.	.	.	Fair	.	.	Fair	Poor
**WHI**									
Stewart [43]	.	Good	.	.	.	.	.	Good	Fair
**VOLP**									
Zhang [23]	.	Good	.	.	.	.	.	Good	.

*WLQ* = *Work Limitations Questionnaire*; *EWPS* = *Endicott Work Productivity Scale*; *WPSI* = *Work Productivity Short Inventory*; *WPAI* = *Work Productivity and Activity Impairment Instrument*; *AMA*-*guide* = *American Medical Association*-*Guides*; *WHO HPQ* = *World Health Organization Health and work Performance Questionnaire*; *SPS* = *Stanford Presenteeism Scale*; *PRODISQ* = *PROductivity and Disease Questionnaire*; *HRPQ*-*D* = *Health*-*Related Productivity Questionnaire Diary*; *HLQ* = *Health and Labor Questionnaire*; *Q&Q* = *Quality and Quantity questionnaire*; *WBA*-*P* = *Well*-*Being Assessment for Productivity*; *HWQ* = *Health and Work Questionnaire*; *WHI* = *Work and Health Interview*; *VOLP* = *Valuation of Lost Productivity questionnaire.*

Table 4 presents the level of evidence for each measurement properties per instrument by synthesising the results per instrument and their accompanying level of evidence. The methodological quality for each instrument and measurement property according to the three main quality domains (reliability, validity and responsiveness) is outlined below, along with a brief description of the aim and the content of the instrument. The synthesis of results per instrument and their accompanying level of evidence are presented in line with the content of Table 4. The evidence for each measurement property is mostly limited; for eight out of 15 instruments at least 50% of the information on measurement properties is lacking.

**Table 4:** Level of evidence per measurement property per instrument

**Instrument and paper**	**Internal consistency**	**Reliability**	**Measurement error**	**Content validity**	**Structural validity**	**Hypotheses testing**	**Cross**-**cultural validity**	**Criterion validity**	**Responsiveness**
**WLQ**[19,21,26-28,36,37,42]	+/-	-	N.A.	++	+++	--	N.A.	+/-	--
**EWPS**[19,29]	?	?	N.A.	N.A.	N.A.	+/-	N.A.	?	?
**WPAI**[19,22,39]	?	?	?	N.A.	N.A.	--	N.A.	--	?
**WPSI**[19,21,31]	?	?	N.A.	?	N.A.	?	N.A.	+/-	?
**AMA**-**guides**[30]	N.A.	?	N.A.	N.A.	N.A.	?	N.A.	?	N.A.
**WHO**-**HPQ**[32,33]	N.A.	-	N.A.	+++	+	+/-	N.A.	+/-	?
**SPS**[34,41]	+++	-	N.A.	-	+++	++	N.A.	++	N.A.
**PRODISQ**[24]	N.A.	N.A.	N.A.	+++	N.A.	N.A.	N.A.	N.A.	N.A.
**HRPQ**-**D**[35]	N.A.	-	N.A.	?	N.A.	?	N.A.	-	-
**HLQ**[20,25]	N.A.	-	N.A.	N.A.	N.A.	N.A.	N.A.	--	N.A.
**Q&Q**[20]	N.A.	-	N.A.	N.A.	N.A.	N.A.	N.A.	-	N.A.
**WBA**-**P**[38]	N.A.	N.A.	N.A.	N.A.	+	N.A.	N.A.	+	N.A.
**HWQ**[40]	+	N.A.	N.A.	N.A.	-	N.A.	N.A.	-	?
**WHI**[43]	N.A.	-	N.A.	N.A.	N.A.	N.A.	N.A.	-	-
**VOLP**[23]	N.A.	+	N.A.	N.A.	N.A.	N.A.	N.A.	-	N.A.

*WLQ* = *Work Limitations Questionnaire*; *EWPS* = *Endicott Work Productivity Scale*; *WPSI* = *Work Productivity Short Inventory*; *WPAI* = *Work Productivity and Activity Impairment Instrument*; *AMA*-*guide* = *American Medical Association*-*Guides*; *WHO HPQ* = *World Health Organization Health and work Performance Questionnaire*; *SPS* = *Stanford Presenteeism Scale*; *PRODISQ* = *PROductivity and Disease Questionnaire*; *HRPQ*-*D* = *Health*-*Related Productivity Questionnaire Diary*; *HLQ* = *Health and Labor Questionnaire*; *Q&Q* = *Quality and Quantity questionnaire*; *WBA*-*P* = *Well*-*Being Assessment for Productivity*; *HWQ* = *Health and Work Questionnaire*; *WHI* = *Work and Health Interview*; *VOLP* = *Valuation of Lost Productivity questionnaire N.A*. = *Not Available*; +++ = *Strong positive level of evidence*; ++ = *Moderate positive level of evidence*; + = *Limited positive level of evidence*; - = *Limited negative level of evidence*; -- = *Moderate negative level of evidence*; +/- = *Conflicting level of evidence*; ? = *Unknown level of evidence due to poor methodological quality.*

#### The Work Limitations Questionnaire (WLQ)

The WLQ measures the degree to which health problems interfere with specific aspects of job performance and the productivity impact of these work limitations. The questionnaire consists of four domains and includes a total of 25 items to be rated on a five-point scale [19,21,26-28,36,37,42].

#### Reliability

Six studies evaluated the internal consistency of the WLQ [19,26,27,36,37,42]. The methodological quality of three studies is poor due to small sample sizes [19,26] and lacking factor analysis to check the unidimensionality of the instrument scale [19,36]. One article is of good methodological quality regarding internal consistency [37]. Although it was not explicitly described, it was possible to deduce how missing items were handled. Furthermore, the authors referred to another study in which factor analysis was performed in a similar population, making it possible to check the unidimensionality of the scale. The paper of Tang, Pitts, Solway & Beaton is of fair methodological quality due to the moderate sample size, and it was unclear whether the factor analysis referred to was appropriate to assess the internal consistency of the WLQ because it was not conducted in a similar population [27]. The last study that evaluated the internal consistency of the WLQ was conducted by Walker, Michaud & Wolfe which is of excellent methodological quality [42]. Sample sizes were adequate, the article clearly described how missing items were handled and the unidimensionality of the scale was checked appropriately. The internal consistency statistic was calculated for each separate WLQ component. Because the findings are conflicting (three studies with poor methodological quality, one of fair, one of good, and one of excellent methodological quality and conflicting findings ranging from moderate (Cα 0.74) to very high (Cα 0.97) Cronbach’s alphas) evidence synthesis of the WLQ resulted in conflicting evidence for internal consistency.

Only one study assessed the reliability [36], which is of fair methodological quality regarding this psychometric property. Although the authors calculated intraclass correlation coefficients (ICC 0.58 – 0.92) and a weighted kappa for continuous and dichotomous scores respectively, it was unclear if patients were stable in the interim period of the measurement. Furthermore, the time interval was not stated, the sample size was moderate and there was no description of how missing items were handled [36]. Evidence synthesis of the WLQ resulted in limited negative evidence for reliability (fair methodological quality and correlation coefficients <0.70).

#### Validity

Three studies examined the content validity [27,36,37]. Lerner et al. [36] reported high validity after assessing whether all items referred to relevant measurement constructs, whether all items were relevant for the study population, and whether all items together comprehensively reflected the construct. The methodological quality of the study is good. Both other studies are of fair methodological quality because the aspects of the construct to be measured were poorly described, and it was not taken into account whether all items referred to relevant aspects of the WLQ [27,37]. Evidence synthesis of the WLQ resulted in moderate positive evidence for content validity.

The structural validity was assessed in two studies [37,42], resulting in good methodological quality. Both studies performed confirmatory factor analysis, which was an appropriate analysis in view of the existing information. Further, the sample size included was adequate, and the way the missing items were handled was either described or could be deduced [37,42]. Evidence synthesis of the WLQ resulted in strong positive evidence for structural validity due to the consistent positive findings in two studies of good methodological quality.

Five studies performed hypotheses testing [19,26-28,36]. Three studies formulated a priori hypotheses. However, the measurement properties of the comparator instrument were not adequately described, and there was no evidence that the comparator could be applied to a similar study population [26-28]. Therefore, the methodological quality regarding hypotheses testing is rated fair in these three studies. Two studies are of poor methodological quality because they did not state clear a priori hypotheses [19,36]. Due to the lack of information on the measurement properties of the comparator instruments, it was unclear what was expected in one study [36]. Evidence synthesis of the WLQ resulted in moderate negative evidence for hypotheses testing (three studies with fair methodological quality and two with poor methodological quality and only 60% of the results were in accordance with the hypotheses).

Criterion validity was evaluated in five studies [19,21,28,36,37]. Three of the studies are rated as having poor methodological quality regarding criterion validity [19,36,37]. In two studies it was not clear whether the criterion used could be considered an adequate gold standard [19,36]. In all three studies, the statistical methods applied were inappropriate to assess criterion validity. Although claiming acceptable criterion validity, two studies failed to determine the calculation of specificity and sensitivity for dichotomous scores and solely mentioned the sensitivity to change (in clinical improvements) related to productivity [19,36]. Lerner, Reed, Massarotti, Wester & Burke [37] did not calculate correlations, or areas under the receiver operating curves, for continuous scores but merely calculated averages. Although Ozminkowski, Goetzel, Chang & Long [21] assumed the criterion used could be considered as a gold standard, no evidence was provided. Therefore, the methodological quality is rated fair. One study [28] reported sufficient evidence on the criterion used to consider an adequate gold standard and applied appropriate statistical methods to assess criterion validity. Therefore the paper is of good methodological quality. Evidence synthesis of the WLQ resulted in conflicting evidence for criterion validity because three studies resulted in poor, one in fair, and one in good methodological quality and not all studies present the degree to which the scores of the instrument are an adequate reflection of a ‘gold standard’.

#### Responsiveness

The responsiveness evaluated by two studies [26,28] is of fair methodological quality because of a poor description of the constructs measured by the comparator instrument [28], a lack of information regarding measurement properties of the comparator instrument [26,28], a vague description of the hypotheses [26] and a moderate sample size [26]. Roy et al. [28] observed low correlation between change scores (0.22 < *r* < 0.41), demonstrating moderate responsiveness. To evaluate responsiveness, Erickson et al. [19] calculated the effect sizes (which were moderate, ranging from 0.36 to 0.73) between change scores based on changes in disease severity. The methodological quality is poor because the statistical method applied is not appropriate to test the hypotheses. Furthermore, the calculation methods for sensitivity and specificity were not determined, but were solely mentioned [19]. Evidence synthesis of the WLQ resulted in moderate negative evidence regarding responsiveness (fair and poor methodological quality and negative results).

There were no methodologically sound studies evaluating measurement error and cross-cultural validity of the WLQ.

#### The Endicott Work Productivity Scale (EWPS)

The EWPS assesses the degree to which a wide variety of mental and medical disorders of persons working in a wide variety of job settings, including self-employment, affect the work functioning of these persons. The EWPS contains 25 items scored on a five-point scale [19,29].

#### Reliability

Internal consistency was studied in two papers that evaluated the measurement properties of the EWPS [19,29]. Both studies are of poor methodological quality because no factor analyses were performed, and sample sizes were small [19,29]. Although the internal consistency estimates show positive results with a Cronbach’s alpha of 0.95 [19] and an internal consistency coefficient of 0.93 [29], evidence synthesis of the EWPS resulted in unknown evidence for internal consistency because only studies of poor methodological quality were available.

Endicott et al. [29] assessed the intraclass correlation coefficient of reliability (total score was 0.92). However, test-retest reliability was only assessed in a limited population including a small sample size (N = 16), resulting in poor methodological quality for the paper [29]. Evidence synthesis of the EWPS resulted in unknown evidence regarding reliability because of the paper’s poor methodological quality.

#### Validity

Hypotheses testing were performed in both papers, though no hypotheses were formulated a priori. However, in Endicott et al. [29] it was possible to deduce what was expected. Although the EWPS showed considerable promise as a sensitive measure for assessing the effects on work performance of various disorders, the instruments and constructs used were poorly described, and, therefore the paper is of fair methodological quality. Erickson et al. [19] confirmed their hypothesized expectations. However, the study is of poor methodological quality because expected differences and directions, and the magnitude of the differences were not stated, making it unclear what was expected. Evidence synthesis resulted in conflicting levels of evidence regarding hypotheses testing (one paper of fair, one of poor methodological quality, and conflicting findings in accordance to the deduced hypotheses).

Both papers are of poor methodological quality for criterion validity because the degree to which the scores of an instruments are an adequate reflection of a reasonable gold standard are unknown in both studies [19,29]. Evidence synthesis of the EWPS resulted in unknown evidence for criterion validity because both studies were of poor methodological quality.

#### Responsiveness

Evidence synthesis of the EWPS resulted in unknown evidence for responsiveness because Erickson et al. [19] calculated the effect sizes between change scores based on changes in disease severity (-0.45). The methodological quality is poor because the statistical method applied is not appropriate to test the hypotheses.

There were no methodologically sound studies evaluating measurement error, content validity, structural validity or cross-cultural validity of the EWPS.

#### The Work Productivity and Activity Impairment Instrument (WPAI)

The WPAI measures the effect of general health and symptom severity on work productivity via six questions to be rated on a five-point scale [19,22,39].

#### Reliability

The evidence synthesis of the WPAI resulted in unknown evidence for internal consistency because Erickson et al. [19] did not apply factor analysis to check for unidimensionality of the instrument scale, and because the sample size was small, resulting in poor methodological quality.

Reliability was studied by Reilly, Zbrozek & Dukes [39]. Although correlation coefficients were calculated (ranging from 0.69 up to 0.95), the measurements were not independent, the time interval was not appropriate, and the test conditions were not similar. Therefore, the paper is of poor methodological quality. The evidence synthesis of the WPAI resulted in unknown evidence for reliability because of the paper’s poor methodological quality.

The study by Reilly, Zbrozek & Dukes [39] included measurement error but because the measurements were not independent and the time interval was not appropriate, the methodological quality is poor. The evidence synthesis of the WPAI resulted in unknown evidence for measurement error due to the study’s poor methodological quality.

#### Validity

Three studies performed hypotheses testing [19,22,39]. Although two studies [22,39] stated a priori hypotheses and expected directions of the differences, the relationships were not always as pronounced as were expected and in both studies only a reference to a study on measurement properties of the comparator instrument was provided, resulting in fair methodological quality. Although it could be deduced what was expected in one study [19], and they confirmed their expectations, the study is of poor methodological quality because expected differences and directions, and the magnitude of the differences were lacking. This study is therefore of poor methodological quality. The evidence synthesis of the WPAI resulted in moderate negative evidence for hypotheses testing (two studies of fair methodological quality and one of poor methodological quality).

Criterion validity was assessed in all papers. The paper of Erickson et al. [19] used other instruments (EWPS and WLQ) as a comparator (significant correlation), which cannot be considered as a reasonable gold standard because the WPAI uses single item scales. Therefore, the paper is of poor methodological quality [19]. The two other papers [22,39] are of fair methodological quality because it was not clear how missing items were handled [22] or whether the criterion used could be considered an adequate gold standard [39]. Correlations between the WPAI and SF-36 measures ranged from 0.20 to 0.52 [39] and from 0.34 up to 0.77 when comparing the WPAI outcomes with health status outcomes from another instrument [22]. The evidence synthesis of the WPAI regarding criterion validity resulted in moderate negative evidence (two studies of fair methodological quality and one of poor methodological quality and correlations <0.70).

#### Responsiveness

Responsiveness over time was evaluated by Erickson et al. [19] by calculating effect sizes (ranging from 0.19 to -0.87) between change scores based on changes in disease severity. The study results therefore in poor methodological quality. Evidence synthesis of the WPAI results in unknown evidence for responsiveness because of the study’s poor methodological quality.

There were no methodologically sound studies evaluating the content validity, structural validity or cross-cultural validity of the WPAI.

#### The Work Productivity Short Inventory (WPSI)

The WPSI assesses the prevalence of medical problems that might influence work productivity based on 22 open questions [19,21,31].

#### Reliability

The internal consistency was studied by Erickson et al. [19], resulting in a Cronbach’s alpha of 0.82. The paper results in poor methodological quality because no factor analyses were performed and sample sizes were small. Evidence synthesis of the WPSI resulted in unknown evidence for internal consistency because of the paper’s poor methodological quality.

Goetzel, Ozminkowski & Long [31] evaluated the reliability of the WPAI but used only one measurement and calculated solely percentage agreement. Therefore, the paper is of poor methodological quality. Evidence synthesis of the WPSI resulted in unknown evidence for reliability because of the study’s poor methodological quality.

#### Validity

The content validity was assessed by Goetzel, Ozminkowski & Long [31], and the paper results in poor methodological quality because there was no information on the degree to which the content of the instrument is an adequate representative of the constructs to be measured. No assessment of whether all items were relevant for the study population occurred, solely a simple description on how the most prevalent conditions in a firm are presented and allow for valid data collection. Evidence synthesis of the WPSI resulted in unknown evidence for content validity because of the study’s poor methodological quality.

Two studies performed hypotheses testing [19,31]. Erickson et al. [19] did not report expected differences and directions, and the magnitude of the differences were not stated, making it unclear what was expected. Goetzel, Ozminkowski & Long [31] did not provide any information on the psychometrics of the comparator instrument and the statistical methods (t-test) were inadequate to test the hypotheses. Both papers result in poor methodological quality. Evidence synthesis of the WPSI resulted in unknown evidence regarding hypotheses testing because of the poor methodological quality of the studies.

Three studies assessed the criterion validity for the WPSI [19,21,31]. The paper of Erickson et al. [19] used a comparator without appropriate evidence that it could be considered a reasonable gold standard. Furthermore, only significance levels were reported, an inadequate method to assess criterion validity. The study of Goetzel, Ozminkowski & Long [31] compared three different versions, varying by recall period, based on coefficient of variation to assess criterion validity (resulting all higher than expected (>10)). As the validation of the WPSI cannot (yet) be empirically confirmed, the method used in this study is inappropriate. Furthermore, no correlation coefficients or area under the receiver operating curve were calculated [31]. Therefore, both papers are of poor methodological quality. Ozminkowski et al. [21] provided some information on the criterion used as a gold standard, but no evidence was provided. All correlations were positive and significantly different from zero, although none exceeded 0.38 in magnitude [21]. The methodological quality is rated fair. Evidence synthesis of the WPSI resulted in conflicting evidence for criterion validity.

#### Responsiveness

The responsiveness was evaluated by an effect size (0.49) between change scores based on changes in disease severity [19] and by significant marginal differences between groups with different conditions [31] making the statistical methods applied inappropriate for the hypotheses to be tested. Therefore, the paper results in poor methodological quality. Evidence synthesis of the WPSI resulted in unknown evidence regarding responsiveness due to the paper’s poor methodological quality.

There were no methodologically sound studies evaluating measurement error, structural validity or cross-cultural validity of the WPSI.

#### The American Medical Association (AMA)-guides

The AMA-guides rate loss of functioning and evaluate workability after injury or illness via fifteen questions rated on a ten-point scale [30].

#### Reliability

Forst, Friedman & Chukwu [30] presents intra-class correlation coefficients comparing impairment raters using the fifth versus the sixth editions of AMA’s-Guides and ranged from 0.65 to 0.77. The paper is of poor methodological quality because the sample size was small (N = 16) and only reliability for each or between two editions was calculated. Evidence synthesis of the AMA-guides resulted in unknown evidence for reliability because of the paper’s poor methodological quality.

#### Validity

Although hypotheses were vague, it was possible to deduce what was expected. However, because the sample size was small and other flaws in the design were presented; e.g. no information was provided regarding the measurement properties of the comparator instrument and the data presented a comparison of two versions of the same instrument, resulted in poor methodological quality of the paper of Forst, Friedman & Chukwu [30] is poor. Evidence synthesis of the AMA-guides therefore resulted in unknown evidence for hypotheses testing because of the study’s poor methodological quality.

Because of the small sample size and the lack of psychometric characteristics of the previous version of the guide, the criterion used cannot be considered an adequate gold standard. Therefore, the methodological quality of the study by Forst, Friedman & Chukwu [30] is poor. Evidence synthesis of the AMA-guides resulted in unknown evidence for criterion validity because of the study’s poor methodological quality.

There were no methodologically sound studies evaluating the internal consistency, measurement error, content validity, structural validity, cross-cultural validity or responsiveness of the AMA-guides.

#### The WHO Health and work Performance Questionnaire (WHO HPQ)

The WHO HPQ uses open questions, divided into three categories, to asses indirect workplace costs of illness by measuring absenteeism and presenteeism and critical incidents [32,33].

#### Reliability

Kessler et al. [32] investigated the reliability (correlation 0.521) but did not report how missing items were handled, did not state the time interval, and did not clearly indicate whether the population was stable during the period of measurement. Therefore, the paper is of fair methodological quality. Evidence synthesis of the WHO HPQ resulted in limited negative evidence for reliability because of the study’s fair methodological quality and correlation <0.70.

#### Validity

Two studies assessed the content validity of the WHO HPQ [32,33]. Although one study did not clearly state the sample size [33], it was possible to assume that all items were relevant for the study population and that the purpose was to reflect on the measurement construct in both studies. Both papers are of good methodological quality. Evidence synthesis of the WHO HPQ resulted in strong positive evidence for content validity (good methodological quality and positive results).

To assess structural validity, Kessler et al. [32] conducted exploratory factor analysis. The overall model fit was excellent (*X*^*2*^ =1.1, *P* = 0.3). However, because it was not clear how missing items were handled, the study is of fair methodological quality. Evidence synthesis of the WHO HPQ resulted in limited positive evidence for structural validity.

Hypotheses testing were performed in both studies [32,33]. In both studies hypotheses were vaguely formulated; however, it was possible to deduce what was expected. In one study the deduced hypotheses appeared obtained [32], while in the other not all results were in accordance with the deduced hypotheses [33]. The studies resulted in fair and poor methodological quality respectively. Evidence synthesis of the WHO HPQ resulted in conflicting evidence for hypotheses testing.

Both studies assessed the criterion validity and specified sensitivity and specificity [32,33]. In one study [33], the criterion used could be considered an adequate ‘gold standard’, and the evidence was provided. However, it could only be deduced how missing items were handled. This study resulted in good methodological quality. Area under the ROC curve calculated ranged from 0.63 to 0.69. The other study [32] reported sensitivity and specificity but did not provide a description of how missing items were handled, and it was unclear whether the gold standard was appropriate. The methodological quality of this study is fair. Evidence synthesis of the WHO HPQ resulted in conflicting evidence for criterion validity.

#### Responsiveness

Because the background of the studies from which the respondents are derived was unclear, and there was lacking information on how missing items were handled, the responsiveness in Kessler et al. [32] results in poor methodological quality. Evidence synthesis resulted in unknown evidence for responsiveness because of the study’s poor methodological quality.

There were no methodologically sound studies evaluating the internal consistency, measurement error, cross-cultural validity or responsiveness of the WHO HPQ.

#### The Stanford Presenteeism Scale (SPS)

The SPS evaluates the impact of health problems on individual performance and productivity by rating six statements on a five-point scale [34,41].

#### Reliability

Two studies assessed the internal consistency of the SPS [34,41]. Both studies performed factor analyses and calculated Cronbach’s alpha; 0.80 and 0.82 respectively. Though there was no description of how missing items were handled, it could be deduced. Therefore, the studies result in good methodological quality. Evidence synthesis of the SPS resulted in strong positive evidence for internal consistency (two studies of good methodological quality and positive findings).

Turpin et al. [41] assessed reliability without stating the time interval, and without describing how missing items were handled. The Pearson correlation coefficient was calculated and indicated a strong negative relationship of -0.60 (P < 0.001). The methodological quality of the study is fair. Evidence synthesis of the SPS resulted in limited negative evidence for reliability.

#### Validity

Because Koopman et al. [34] solely assessed discriminative validity for a scale, where none of the relationships showed a strong degree of magnitude, and did not assess whether all items were relevant for the purpose of the instrument. The methodological quality of the study is fair and evidence synthesis of the SPS resulted in limited negative evidence for content validity.

In both studies, (classical) factor analysis was conducted to assess structural validity [34,41], and indicated that the instrument captured the dimensions intended to asses, providing positive evidence for structural validity. The type of factor analysis was appropriate in view of the existing information and the way missing items were handled could be deduced. Therefore, the studies are of good methodological quality. Evidence synthesis of the SPS resulted in strong positive evidence for structural validity (two studies of good methodological quality and positive findings).

Because hypotheses were vaguely formulated, the comparator instrument was the long version of the instrument under evaluation, and information on the measurement properties of the comparator were lacking, [34] is fair. The methodological quality for the study by Turpin et al. [41] is also rated fair because solely a number of hypotheses were formulated a priori and the comparator instrument measured another construct (work output versus work impairment). Therefore, one cannot be sure whether the measurement properties of the comparator instrument apply to this study population. Evidence synthesis of the SPS resulted in moderate positive evidence for hypotheses testing (two studies of fair methodological quality and the results were in accordance with the hypotheses).

Both studies assessed criterion validity but did not provide the percentage of missing items [34,41]. Additionally, Koopman et al. [34] did not provide evidence but only assumed that the criterion used could be considered an adequate gold standard. The continuous scores on both instruments correlated strongly. To assess criterion validity Turpin et al. [41] calculated correlations between SPS metrics and comparator dimensions which were in the expected direction and mostly significantly different from zero. However, Turpin et al. [41] did not make it clear how missing items were handled. Evidence synthesis of the SPS resulted in moderate positive evidence for criterion validity (fair methodological quality and positive findings).

There were no methodologically sound studies evaluating measurement error, cross-cultural validity or responsiveness of the SPS.

#### The Productivity and Disease Questionnaire (PRODISQ)

In brief, the PRODISQ measures and valuates productivity costs by assessing the relationship between health and productivity based on open and multiple-choice questions in seven modules [24].

#### Validity

Koopmanschap et al. [24] assessed whether all items refer to relevant aspects of the constructs to be measured, assessed if all items were relevant to the different study populations, and whether the items were relevant for the application purpose (positive item relevance). Furthermore, the authors assessed whether all items together comprehensively reflected the constructs to be measured (positive item comprehensiveness). Evidence synthesis of the PRODISQ resulted in strong positive evidence for content validity (excellent methodological quality and positive findings).

There were no methodologically sound studies evaluating the internal consistency, reliability, measurement error, structural validity, hypotheses testing, cross-cultural validity or responsiveness of the PRODISQ.

#### The Health-Related Productivity Questionnaire Diary (HRPQ-D)

The HRPQ-D measures work productivity data related to health-related labour force participation via nine open and multiple-choice questions [35].

#### Reliability

Kumar et al. [35] investigated the reliability by calculating correlation coefficients (generally non-statistically significant, ranging from -0.449 up to 0.806) between the reported productivity loss data and the symptom scores of repeated measurements within individuals but did not provide evidence as to whether systematic change had occurred. Additionally, several methodological flaws in the design and execution of the study, such as sampling bias, a lack of evidence that the patients were stable and different scoring systems for symptom severity resulted in the study being of fair methodological quality. Evidence synthesis of the HRPQ-D resulted in limited negative evidence for reliability.

#### Validity

Kumar et al. [35] did not assess whether all items were relevant for the different target populations or whether all items together comprehensively reflected the construct to be measured. The study is therefore of poor methodological quality. Evidence synthesis of the HRPQ-D resulted in unknown evidence for content validity.

A priori hypotheses were vaguely formulated, the expected direction and magnitude of the correlations were poorly described, and no information on the measurement properties of the comparator instrument was given. Generally, the correlations were not statistically significant. The study of Kumar et al. [35] results in poor methodological quality and evidence synthesis of the HRPQ-D resulted in unknown evidence for hypotheses testing.

Kumar et al. [35] did not elaborate on how missing items were handled, and it was unclear whether the criterion used could be considered an adequate gold standard. Therefore, the study results in fair methodological quality. Evidence synthesis of the HRPQ-D resulted in limited negative evidence for criterion validity (fair methodological quality and negative findings).

#### Responsiveness

Hypotheses were vague, neither magnitudes nor directions of the correlations were stated a priori, and information on the psychometrics of the comparator was lacking. Evaluating the instrument’s responsiveness to change yielded positive findings. However, the significant correlation values ranged from -0.161 to 0.422 [35]. Evidence synthesis of the HRPQ-D resulted in limited negative evidence for responsiveness (fair methodological quality and negative findings).

There were no methodologically sound studies evaluating the internal consistency, measurement error, structural validity or cross-cultural validity of the HRPQ-D.

#### The Health and Labour Questionnaire (HLQ)

The HLQ collects data on the relationship between illness and treatment and work performance. The HLQ is a modular questionnaire with four modules and response options on a four-point scale [20,25].

#### Reliability

In the study of Meerding et al. [20], inter-rater reliability was assessed. However, because there was no clear description of how missing items were handled and it was only assumable that participants and test conditions were stable in the period on the construct to be measured, the methodological quality of the study is fair. The agreement on self-reported productivity loss due to health problems showed a poor κ-value of 0.18 [20]. Evidence synthesis of the HLQ resulted in limited negative evidence for reliability.

#### Validity

Two studies assessed the HLQ’s criterion validity [20,25]. Both studies calculated correlation coefficients (significant correlations ranging from 0.33 to 0.73 [20] and Pearson correlation between 0.41 and 0.56 [25]) However, it was unclear for the study conducted by Van Roijen, Essink-Bot, Koopmanschap, Bonsel & Rutten [25] whether the criterion used could be considered as a gold standard. Meerding et al. [20] lacked information on how missing items were handled. Therefore, the methodological quality of both studies is fair. Evidence synthesis of the HLQ resulted in moderate negative evidence for criterion validity because of the fair methodological quality of the studies and negative findings (correlations <0.70).

There were no methodologically sound studies evaluating the internal consistency, measurement error, content validity, structural validity, hypotheses testing, cross-cultural validity or responsiveness of the HLQ.

#### The Quality and Quantity questionnaire (Q&Q)

The Q&Q questionnaire scores two questions about the quantity and quality of the work performed on the last working day on a 10-point scale [20].

#### Reliability

Meerding, Ijzelenberg, Koopmanschap, Severens & Burdorf [20] did not provide a clear description of how missing items were handled. The stability of participants and test conditions in the period on the construct to be measured could be deduced. The methodological quality of the study is fair. An unweighted kappa (κ-value = 0.18) was calculated to assess the inter-rater reliability [20]. Evidence synthesis of the Q&Q resulted in limited negative evidence for reliability.

#### Validity

Although the criterion used could be considered an adequate gold standard based on the evidence provided, Meerding, Ijzelenberg, Koopmanschap, Severens & Burdorf [20] lacked a clear description of how missing items were handled. Self-reported productivity measured with the QQ correlated significantly with objective work output, however the strength of the correlation (*r* =0.48) was poor [20]. Evidence synthesis of the Q&Q resulted in limited negative evidence for criterion validity.

There were no methodologically sound studies evaluating the internal consistency, measurement error, content validity, structural validity, hypotheses testing, cross-cultural validity or responsiveness of the Q&Q.

#### The Well-Being Assessment for Productivity (WBA-P)

The WBA-P provides an evaluation of job performance loss due to wellbeing-related barriers. The barriers are based on twelve items to be rated from zero to 100 [38].

#### Validity

Prochaska et al. [38] performed factor analysis to assess the structural validity but did not describe the percentage of missing items. Because it could be deduced how missing items were handled, the methodological quality of the study is good. A hierarchical two-factor model demonstrated good fit (*X*^*2*^ = 544.34) with acceptable internal consistency on the subscales (Cronbach’s alpha 0.73 – 0.83) [38]. Evidence synthesis of the WBA-P resulted in limited positive evidence for structural validity because of the paper’s good methodological quality.

Prochaska et al. [38] assessed the criterion validity by calculating correlations and multivariate variance analysis. Although no evidence was provided, it was stated that the criterion used could be considered an adequate gold standard. Significant interactions between the WBA-P and the comparator instrument existed [38]. Evidence synthesis of the WBA-P resulted in limited positive evidence for structural validity (good methodological quality and positive findings).

There were no methodologically sound studies evaluating the internal consistency, reliability, measurement error, content validity, hypotheses testing, cross-cultural validity or responsiveness of the WBA-P.

#### The Health and Work Questionnaire (HWQ)

The HWQ measures workplace productivity and workers’ health multidimensionally via 24 questions to be rated on a ten-point scale [40].

#### Reliability

Shikiar, Halpern, Rentz & Khan [40] conducted factor analysis to check the unidimensionality of the scale. The factor analysis resulted in six factors with eigenvalues greater than 1.0 and accounted for 69% of the variance in the correlation matrix. The sample size was adequate (N = 294), and Cronbach’s coefficient alpha’s were calculated to assess the internal consistency for each subscale separately (ranging from 0.72 to 0.96). The percentage of missing items was not described [40]. The methodological quality of the study is rated fair because it was not clear how missing items were handled. Evidence synthesis of the HWQ resulted in limited positive evidence for internal consistency (fair methodological quality and positive findings: correlations >0.70).

#### Validity

Shikiar, Halpern, Rentz & Khan [40] assessed the structural validity by conducting a principal components factor analysis with orthogonal rotation confirming that the items on the HWQ are measuring several different dimensions related to productivity and the items on the HWQ do not ‘hang together’ as well as the items on the individual scales [40]. Because it was not clear how missing items were handled, the methodological quality of the study is fair. Evidence synthesis of the HWQ resulted in limited negative evidence for structural validity.

In assessing the criterion validity Shikiar, Halpern, Rentz & Khan [40] did not make it clear how missing items were handled nor provided evidence, although it could be deduced, that the criterion used could be considered an adequate gold standard. Correlations with two objective productivity measures ranged from -0.043 to -0.219 and certain subscales did not significantly correlate with overall productivity. Evidence synthesis of the HWQ resulted in limited negative evidence for criterion validity (fair methodological quality and weak correlations).

#### Responsiveness

Shikiar, Halpern, Rentz & Khan [40] lacked a priori information on the measurement properties of the comparator instrument, the expected direction and magnitude of the correlation. The methodological quality of the study is poor. Evidence synthesis of the HWQ resulted in unknown evidence for responsiveness.

There were no methodologically sound studies evaluating the reliability, measurement error, content validity, hypotheses testing or cross-cultural validity of the HWQ.

#### The Work and Health Interview (WHI)

In summary, the WHI captures data on absence time from work, reduced performance, and health-related causes based on a six-module, computer-assisted telephone interview [43].

#### Reliability

Stewart, Ricci, Leotta & Chee [43] studied reliability whereby independent measurements with an appropriate time interval were conducted. Both Pearson’s (0.59) and Spearman’s (0.63) correlation coefficients were derived between two measures. Evidence was provided that test conditions were similar, it was described how missing items were handled and the sample size (N = 66) was adequate [43]. Evidence synthesis of the WHI resulted in limited negative level of evidence for reliability because of the study’s good methodological quality and relatively negative findings (correlations <0.70).

#### Validity

In the study conducted by Stewart, Ricci, Leotta & Chee [43], it could easily be assumed that the criterion used (diary work time measures) could be considered an adequate gold standard. The Pearson’s (Spearman’s) correlation between the WHI and diary total productivity time measure was 0.50 (0.46) and there were no other important methodological flaws in the design of the study [43]. Evidence synthesis of the WHI resulted in limited negative evidence for reliability (good methodological quality and negative findings).

#### Responsiveness

Hypotheses were vaguely formulated and limited evidence on the measurement properties of the comparator instrument was available. The methodological quality of the study is fair. The correlations between change scores of time not working at work (continuous scores regarding performance data) were (Pearson’s correlation) 0.19 and (Spearman’s) 0.33 [43] Evidence synthesis of the WHI resulted in limited negative evidence for responsiveness.

There were no methodologically sound studies evaluating the internal consistency, measurement error, content validity, structural validity, hypotheses testing or cross-cultural validity of the WHI.

#### The Valuations of Lost Productivity questionnaire (VOLP)

The VOLP assesses labour input loss due to health via 36 open and multiple-choice questions in six sections [23].

#### Reliability

Zhang, Bansback, Kopec & Anis [23] assessed reliability over time by means of a test-retest analysis. Kappa statistics were calculated, all being statistically significant and reporting adequate agreement. Due to the time frame (2 weeks), the type of administration (postal questionnaire) and the environment (at home), it could be assumed that the test conditions were similar. The methodological quality of the study is good regarding test-retest reliability. Evidence synthesis of the VOLP resulted in limited positive evidence for reliability.

#### Validity

In order to assess the constructs of the VOLP, the criterion used could be considered as a reasonable gold standard because evidence regarding the psychometric properties of the comparators is provided in the study by Zhang Zhang, Bansback, Kopec & Anis [23]. There is no description on how missing items were handled; however, it could be deduced. Furthermore, Spearman’s correlation coefficients were calculated to assess criterion validity. The correlations between the VOLP outcomes and corresponding WPAI outcomes ranged from 0.39 to 0.57. The methodological quality of the paper is good and thus the evidence synthesis of the VOLP resulted in limited negative evidence for reliability (good methodological quality and Spearman correlations <0.7).

There were no methodologically sound studies evaluating the internal consistency, measurement error, content validity, structural validity, hypotheses testing, cross-cultural validity or responsiveness of the VOLP.

## Conclusions and discussion

Twenty-five studies on measurement properties of 15 generic self-reported instruments measuring health-related productivity changes have been systematically reviewed, and their methodological quality has been evaluated using the COSMIN-checklist in a best evidence synthesis. The WLQ is the most frequently evaluated instrument. Structural validity and content validity reported a strong and moderate positive level of evidence respectively. For measurement error and cross-cultural validity, no information was available and the internal consistency and criterion validity resulted in conflicting evidence. Reliability, hypotheses testing and responsiveness resulted in limited negative and moderate negative evidence respectively. Due to poor methodological quality, the EWPS, WPAI, and WPSI showed unknown levels of evidence for almost half of the information on measurement properties. For eight questionnaires (AMA-guides, PRODISQ, HLQ, Q&Q, WBA-P, HWQ, WHI, VOLP) at least half of the information on measurement properties per questionnaire was lacking. Four instruments (WLQ, WHO-HPQ, SPS, and PRODISQ) showed strong or moderate positive levels of evidence for some of the measurement properties.

The main strength of most studies was that they reported detailed information regarding the population characteristics, sampling methods, the setting and the country where the studies were conducted.

There were, however, many limitations. First, the generalisability of the results of the studies on measurement properties was low, mainly because of selective samples, the non-reporting of and the lack of information regarding the handling of missing values, and inadequate sample sizes.

Second, most studies recruited convenience samples, which might not cover the entire target population [44]. Ozminkanski et al. was the only study that consecutively recruited workers with job-related accidents or injuries, which could be a reasonable representation of the workers with lost productivity at the workplace [21].

Third, although Zhang and colleagues examined the measurement properties in two countries (UK and Ireland), no international samples demonstrated the cross-cultural validity of their measures [22,23]. Most studies were conducted in the United States, which makes it difficult to discern whether the instruments are appropriate for study populations outside of the United States. The results of this review emphasize the need for international studies on measurement properties as well as additional evaluation studies conducted worldwide to examine the cross-cultural appropriateness of these measures to improve generalizability. The Work Role Functioning Questionnaire (WRFQ) measures perceived difficulties in meeting work demands among employees given their physical and emotional problems. The WRFQ addresses work outcomes in an attempt to describe how health affects work role functioning [45-48]. Despite the fact that the WRFQ is to be used as a detection instrument to identify, and not value decreased productivity, it can serve as an excellent example since several studies have translated and adapted the WRFQ to Canadian French [45], Brazilian Portuguese [46], Dutch [47], and Spanish [48]. These studies demonstrate a systematic procedure for translation and cross-cultural adaptation which can serve as excellent examples for future studies attempting to adapt and validate instruments in other cultures.

Fourth, almost half of the reviewed studies reported item and unit nonresponses under 50%, which might indicate selection bias, further hampering the generalizability of the results [44]. Inadequate descriptions of the handling of missing values might suggest non-random missing items, which could bias the results and lead to misinterpretation and misjudgement of the measurement properties of an instrument. Furthermore, if missing values are inappropriately handled, bias in parameter estimates can occur, resulting in lower samples sizes and thus lower statistical power. High percentages of missing values on specific items might even indicate that an item is not relevant for the study population, perhaps pointing to ambiguous formulations and hampering the validity of the instruments [17,49]. In light of the flaws presented from previous studies, response rates should be accurately reported, including information on the handling of missing items, and if randomness of nonresponse occurred, it should be examined and reported in future studies.

Fifth, based on the results of this systematic review it can be concluded that the information regarding the measurement properties of generic self-reported instruments measuring health-related productivity changes is mostly limited or of poor to fair methodological quality. The results should be treated with caution due to the missing information on the remaining measurement properties. Especially when considering measurement error and cross-cultural validity, wherefore (almost) no information was available.

Sixth, although it is difficult to determine the criterion validity without a real gold standard for health-related productivity change instruments, most studies considered the extent to which scores on the instrument of interest could be adequately reflected to a predetermined comparator. Criterion validity is therefore most frequently evaluated, but only five studies on this measurement property were of good methodological quality. As a consequence, the SPS and the WBA-P yielded moderate and limited positive levels of evidence for criterion validity respectively.

Seventh, it is difficult to determine the responsiveness of the different health-related productivity change instruments because almost all of the retrieved studies were of poor or fair methodological quality regarding responsiveness. Because the instruments are often used as an outcome measure to determine the costs of lost productivity, specific hypotheses regarding expected correlations with other constructs must be formulated a priori when developing a new measure.

Eighth, the internal consistency statistic only gets an interpretable meaning when the interrelatedness among the items is determined as a set of items that together form a reflective model. The internal consistency of an instrument is thus reflected in the quality assessment of structural validity, and vice versa. If the structural validity was not assessed by analysing the unidimensionality (there is no evidence that the scales are unidimensional), no internal consistency statistic can be properly interpreted. Four studies resulted in good methodological quality on structural validity for the WLQ and SPS, and also in good internal consistency for both instruments. Most of the instruments with unknown levels of evidence due to poor methodological quality regarding internal consistency also lacked information on structural validity.

Ultimately, some general issues on measuring productivity changes should be addressed. First, the concept of productivity loss due to illness is, according to economic theory, based on the concept of a production function where output is a function of capital input, labour input and technology. The focus of most productivity measurement instruments, as has been seen, is on the individual’s labour input; measuring the time a person is not at work due to health complaints (absenteeism), or is not productive while at work due to health complaints (presenteeism). However, job and workplace characteristics also play a key role and differ among countries, which are reflected in the socio-political context in which the study takes place. For example, in some countries that have a workers’ compensation system, such as Canada and the United States, there is a differentiation between work and non-work related disability. In other countries, such as the Netherlands, no such differentiation exists. Due to these variations arising from the social-political context, one cannot assume a ‘one size fits all’ mentality when comparing instrument effectiveness across countries or cultures. Transparency in reporting the key aspects of measurement and validation of health-related productivity would simplify the comparability and usability of the results for occupational and health economic decision making.

Another point to be addressed is that although the COSMIN taxonomy might contribute to a better understanding in the terminology used in validation studies and provides a structured procedure for the evaluation of the methodological quality of the studies on measurement properties, the taxonomy provides a lot of room for interpretation in the checklist items. To minimize interpretability differences between reviewers (CYGN, AER, SE), decisions had to be made on how to score the different items. For example, a problem encountered during the rating of ‘criterion validity’ was the absence of a gold standard in health-related productivity change instruments. One example of how this problem was dealt with was by assuming the original long version of the shortened instrument being assessed was an adequate comparator, and can thus be seen as a ‘gold standard’. Furthermore, since the studies were systematically reviewed on the measurement properties of self-reported instruments which encounter subjective data, it was agreed that objective, registered data could serve as an adequate comparator as well. Predetermined and transparent arguments that the gold standard is ‘gold’ had to be thoroughly discussed and decided beforehand to assess criterion validity.

Finally, although an agreement was reached that objective data could be seen as a ‘gold standard’ for collecting lost productivity data it should be in mind that both objective and self-reported instruments have their advantages and disadvantages, which need to be weighed per research question. For example, when using objective insurance data, a particular disadvantage is that the data reflects what has been compensated. What has been compensated does not necessarily reflect the actual time a worker has been unable to work. Productivity changes related to sick leave should therefore always be supplemented by the productivity changes due to decreased work performance; i.e. presenteeism, to avoid underestimations.

### Recommendations

Although only cautious advice can be provided on the most appropriate instruments to capture changes in productivity for use in occupational and economic health practice, the WLQ is cautiously recommended at the moment because the instrument is most frequently evaluated and moderate respectively strong positive evidence was found for content and structural validity respectively. However, negative evidence was found for reliability, hypothesis testing and responsiveness. The WLQ is only used in an English-speaking study population. Using the PRODISQ is cautiously preferred when conducting a study in the Netherlands based on its strong positive evidence for content validity, although evidence for the other measurement properties is lacking. In order to improve the interpretation of the PRODISQ scores, more research regarding the measurement properties (aside from content validity) is needed. The Stanford Presenteeism Scale (SPS) can also be cautiously recommended as it is evaluated in two studies and showed strong positive results for internal consistency and structural validity, and moderate positive results for hypotheses testing and criterion validity. Limited negative evidence however was available for reliability and content validity and information on the other measurement properties was lacking.

Better knowledge and usage of key methodological principles based on quality checklists, such as COSMIN, is recommended to provide high-quality studies evaluating the measurement properties of new and existing instruments in the future. High-quality studies that evaluate and provide strong evidence for the unknown measurement properties, especially cross-cultural validity, are recommended to improve the generalizability and applicability of generic self-reported health-related productivity change instruments. Given the large number of available productivity instruments the development of new instruments is not recommended, but rather improvement of the existing instruments.

## Abbreviations

AMA-guide: American Medical Association-Guides; COSMIN: Consensus-based Standards for the selection of health Measurement Instruments; EWPS: Endicott Work Productivity Scale; HLQ: Health and Labor Questionnaire; HRPQ-D: Health-Related Productivity Questionnaire Diary; HWQ: Health and Work Questionnaire; SPS: Stanford Presenteeism Scale; PRODISQ: PROductivity and Disease Questionnaire; Q&Q: Quality and Quantity questionnaire; VOLP: Valuation of Lost Productivity questionnaire; WBA-P: Well-Being Assessment for Productivity; WHI: Work and Health Interview; WHO HPQ: World Health Organization Health and work Performance Questionnaire; WLQ: Work Limitations Questionnaire; WPAI: Work Productivity and Activity Impairment Instrument; WPSI: Work Productivity Short Inventory.

## Competing interests

The authors declare that they have no competing interests.

## Authors’ contributions

All authors (CYGN, SMAAE, FJN, AER) made substantial contributions to conception and design, and analysis and interpretation of the data. All authors have been involved in drafting the manuscript and revised it critically for important intellectual content. Three reviewers determined the methodological quality of the studies (CYGN, AER, SMAAE). Consensus on the methodological quality of the studies and the evidence synthesis was reached through discussion (CYGN, SMAAE, FJN, AER). All authors have given their final approval of the version to be published.

## Pre-publication history

The pre-publication history for this paper can be accessed here:

http://www.biomedcentral.com/1471-2458/14/115/prepub

## Supplementary Material

Additional file 1: Table S1 Search strategy. Click here for file

Additional file 2: Table S2 Detailed description of the generic self-reported instruments measuring health-related productivity changes. Click here for file

## References

[B1] Lambeek LC Van Tulder MW Swinkels IC Koppes LL Anema JR Van Mechelen W The trend in total cost of back pain in The Netherlands in the period 2002 to 2007 Spine (Phila Pa 1976) 2011 36 1050 1058 10.1097/BRS.0b013e3181e70488 21150697

[B2] Stock S Redaelli M Luengen M Wendland G Civello D Lauterbach KW Asthma: prevalence and cost of illness Eur Respir J 2005 25 47 53 10.1183/09031936.04.00116203 15640322

[B3] Van Tulder MW Koes BW Bouter LM A cost-of-illness study of back pain in The Netherlands Pain 1995 62 233 240 10.1016/0304-3959(94)00272-G 8545149

[B4] Goetzel RZ Hawkins K Ozminkowski RJ Wang S The health and productivity cost burden of the "top 10" physical and mental health conditions affecting six large U.S. employers in 1999 J Occup Environ Med 2003 45 5 14 10.1097/00043764-200301000-00007 12553174

[B5] Drummond M McGuire A Economic Evaluation in Helath Care Merging theory with practice 2001 New York: Oxford University Press

[B6] Schultz AB Chen CY Edington DW The cost and impact of health conditions on presenteeism to employers: a review of the literature Pharmacoeconomics 2009 27 365 378 10.2165/00019053-200927050-00002 19586075

[B7] Smit F Willemse G Koopmanschap M Onrust S Cuijpers P Beekman A Cost-effectiveness of preventing depression in primary care patients: randomised trial Br J Psychiatry 2006 188 330 336 10.1192/bjp.188.4.330 16582059

[B8] Brouwer WB Koopmanschap MA Rutten FF Productivity losses without absence: measurement validation and empirical evidence Health Policy 1999 48 13 27 10.1016/S0168-8510(99)00028-7 10539583

[B9] Hoeijenbos M Bekkering T Lamers L Hendriks E Van Tulder M Koopmanschap M Cost-effectiveness of an active implementation strategy for the Dutch physiotherapy guideline for low back pain Health Policy 2005 75 85 98 10.1016/j.healthpol.2005.02.008 16298231

[B10] Lotters F Meerding WJ Burdorf A Reduced productivity after sickness absence due to musculoskeletal disorders and its relation to health outcomes Scand J Work Environ Health 2005 31 367 374 10.5271/sjweh.920 16273963

[B11] Pauly MV Nicholson S Polsky D Berger ML Sharda C Valuing reductions in on-the-job illness: 'presenteeism' from managerial and economic perspectives Health economics 2008 17 469 485 10.1002/hec.1266 17628862

[B12] Lofland JH Pizzi L Frick KD A review of health-related workplace productivity loss instruments Pharmacoeconomics 2004 22 165 184 10.2165/00019053-200422030-00003 14871164

[B13] Prasad M Wahlqvist P Shikiar R Shih YC A review of self-report instruments measuring health-related work productivity: a patient-reported outcomes perspective Pharmacoeconomics 2004 22 225 244 10.2165/00019053-200422040-00002 14974873

[B14] Terwee CB Mokkink LB Knol DL Ostelo RW Bouter LM De Vet HC Rating the methodological quality in systematic reviews of studies on measurement properties: a scoring system for the COSMIN checklist Qual Life Res 2011 21 651 657 21732199 10.1007/s11136-011-9960-1 PMC3323819

[B15] Terwee CB Jansma EP Riphagen II De Vet HC Development of a methodological PubMed search filter for finding studies on measurement properties of measurement instruments Qual Life Res 2009 18 1115 1123 10.1007/s11136-009-9528-5 19711195 PMC2744791

[B16] Mokkink LB Terwee CB Patrick DL Alonso J Stratford PW Knol DL Bouter LM De Vet HC The COSMIN study reached international consensus on taxonomy, terminology, and definitions of measurement properties for health-related patient-reported outcomes J Clin Epidemiol 2010 63 737 745 10.1016/j.jclinepi.2010.02.006 20494804

[B17] Mokkink LB Terwee CB Patrick DL Alonso J Stratford PW Knol DL Bouter LM De Vet HC The COSMIN checklist for assessing the methodological quality of studies on measurement properties of health status measurement instruments: an international Delphi study Qual Life Res 2010 19 539 549 10.1007/s11136-010-9606-8 20169472 PMC2852520

[B18] Furlan AD Pennick V Bombardier C Van Tulder M Editorial Board CBRG. 2009 updated method guidelines for systematic reviews in the Cochrane Back Review Group Spine (Phila Pa 1976) 2009 34 1929 1941 10.1097/BRS.0b013e3181b1c99f 19680101

[B19] Erickson SR Guthrie S VanEtten-Lee M Himle J Hoffman J Santos SF Janeck AS Zivin K Abelson JL Severity of anxiety and work-related outcomes of patients with anxiety disorders Depress Anxiety 2009 26 1165 1171 10.1002/da.20624 19842165

[B20] Meerding WJ IJ W Koopmanschap MA Severens JL Burdorf A Health problems lead to considerable productivity loss at work among workers with high physical load jobs J Clin Epidemiol 2005 58 517 523 10.1016/j.jclinepi.2004.06.016 15845339

[B21] Ozminkowski RJ Goetzel RZ Chang S Long S The application of two health and productivity instruments at a large employer J Occup Environ Med 2004 46 635 648 10.1097/01.jom.0000131797.52458.c8 15247802

[B22] Zhang W Bansback N Boonen A Young A Singh A Anis AH Validity of the work productivity and activity impairment questionnaire–general health version in patients with rheumatoid arthritis Arthritis Res Ther 2010 12 R177 10.1186/ar3141 20860837 PMC2991008

[B23] Zhang W Bansback N Kopec J Anis AH Measuring time input loss among patients with rheumatoid arthritis: validity and reliability of the Valuation of Lost Productivity questionnaire J Occup Environ Med 2011 53 530 536 10.1097/JOM.0b013e318218abf1 21508868

[B24] Koopmanschap MA PRODISQ: a modular questionnaire on productivity and disease for economic evaluation studies Expert Rev Pharmacoecon Outcomes Res 2005 5 23 28 10.1586/14737167.5.1.23 19807557

[B25] Van Roijen L Essink-Bot ML Koopmanschap MA Bonsel G Rutten FF Labor and health status in economic evaluation of health care. The Health and Labor Questionnaire Int J Technol Assess Health Care 1996 12 405 415 10.1017/S0266462300009764 8840661

[B26] Beaton DE Kennedy CA Beyond return to work: testing a measure of at-work disability in workers with musculoskeletal pain Qual Life Res 2005 14 1869 1879 10.1007/s11136-005-3865-9 16155774

[B27] Tang K Pitts S Solway S Beaton D Comparison of the psychometric properties of four at-work disability measures in workers with shoulder or elbow disorders J Occup Rehabil 2009 19 142 154 10.1007/s10926-009-9171-6 19301108

[B28] Roy JS MacDermid JC Amick BC 3rd Shannon HS McMurtry R Roth JH Grewal R Tang K Beaton D Validity and responsiveness of presenteeism scales in chronic work-related upper-extremity disorders Phys Ther 2011 91 254 266 10.2522/ptj.20090274 21212376

[B29] Endicott J Nee J Endicott Work Productivity Scale (EWPS): a new measure to assess treatment effects Psychopharmacol Bull 1997 33 13 16 9133746

[B30] Forst L Friedman L Chukwu A Reliability of the AMA Guides to the Evaluation of Permanent Impairment J Occup Environ Med 2010 52 1201 1203 10.1097/JOM.0b013e3181fd2782 21124242

[B31] Goetzel RZ Ozminkowski RJ Long SR Development and reliability analysis of the Work Productivity Short Inventory (WPSI) instrument measuring employee health and productivity J Occup Environ Med 2003 45 743 762 10.1097/01.jom.0000079085.95532.32 12855915

[B32] Kessler RC Ames M Hymel PA Loeppke R McKenas DK Richling DE Stang PE Ustun TB Using the World Health Organization Health and Work Performance Questionnaire (HPQ) to evaluate the indirect workplace costs of illness J Occup Environ Med 2004 46 S23 S37 10.1097/01.jom.0000126683.75201.c5 15194893

[B33] Kessler RC Barber C Beck A Berglund P Cleary PD McKenas D Pronk N Simon G Stang P Ustun TB Wang P The World Health Organization Health and Work Performance Questionnaire (HPQ) J Occup Environ Med 2003 45 156 174 10.1097/01.jom.0000052967.43131.51 12625231

[B34] Koopman C Pelletier KR Murray JF Sharda CE Berger ML Turpin RS Hackleman P Gibson P Holmes DM Bendel T Stanford presenteeism scale: health status and employee productivity J Occup Environ Med 2002 44 14 20 10.1097/00043764-200201000-00004 11802460

[B35] Kumar RN Hass SL Li JZ Nickens DJ Daenzer CL Wathen LK Validation of the Health-Related Productivity Questionnaire Diary (HRPQ-D) on a sample of patients with infectious mononucleosis: results from a phase 1 multicenter clinical trial J Occup Environ Med 2003 45 899 907 10.1097/01.jom.0000083039.56116.79 12915792

[B36] Lerner D Amick BC 3rd Rogers WH Malspeis S Bungay K Cynn D The Work Limitations Questionnaire Med Care 2001 39 72 85 10.1097/00005650-200101000-00009 11176545

[B37] Lerner D Reed JI Massarotti E Wester LM Burke TA The Work Limitations Questionnaire's validity and reliability among patients with osteoarthritis J Clin Epidemiol 2002 55 197 208 10.1016/S0895-4356(01)00424-3 11809359

[B38] Prochaska JO Evers KE Johnson JL Castle PH Prochaska JM Sears LE Rula EY Pope JE The well-being assessment for productivity: a well-being approach to presenteeism J Occup Environ Med 2011 53 735 742 10.1097/JOM.0b013e318222af48 21691220

[B39] Reilly MC Zbrozek AS Dukes EM The validity and reproducibility of a work productivity and activity impairment instrument Pharmacoeconomics 1993 4 353 365 10.2165/00019053-199304050-00006 10146874

[B40] Shikiar R Halpern MT Rentz AM Khan ZM Development of the Health and Work Questionnaire (HWQ): An instrument for assessing workplace productivity in relation to worker health Work 2004 22 219 229 15156087

[B41] Turpin RS Ozminkowski RJ Sharda CE Collins JJ Berger ML Billotti GM Baase CM Olson MJ Nicholson S Reliability and validity of the Stanford Presenteeism Scale J Occup Environ Med 2004 46 1123 1133 10.1097/01.jom.0000144999.35675.a0 15534499

[B42] Walker N Michaud K Wolfe F Work limitations among working persons with rheumatoid arthritis: results, reliability, and validity of the work limitations questionnaire in 836 patients J Rheumatol 2005 32 1006 1012 15940759

[B43] Stewart WF Ricci JA Leotta C Chee E Validation of the work and health interview Pharmacoeconomics 2004 22 1127 1140 10.2165/00019053-200422170-00003 15612831

[B44] Carley S Lecky F Statistical consideration for research Emerg Med J 2003 20 258 262 10.1136/emj.20.3.258 12748144 PMC1726086

[B45] Durand MJ Vachon B Hong QN Imbeau D Amick BC 3rd Loisel P The cross-cultural adaptation of the Work Role Functioning Questionnaire in Canadian French Int J Rehabil Res 2004 27 261 268 10.1097/00004356-200412000-00002 15572988

[B46] Gallasch CH Alexandre NM Amick B 3rd Cross-cultural adaptation, reliability, and validity of the work role functioning questionnaire to Brazilian Portuguese J Occup Rehabil 2007 17 701 711 10.1007/s10926-007-9103-2 17909949

[B47] Abma FI Amick BC 3rd Brouwer S van der Klink JJ Bultmann U The cross-cultural adaptation of the work role functioning questionnaire to Dutch Work 2012 43 203 210 22927615 10.3233/WOR-2012-1362

[B48] Ramada JM Serra C Amick BC 3rd Castano JR Delclos GL Cross-cultural adaptation of the Work Role Functioning Questionnaire to Spanish spoken in Spain J Occup Rehabil 2013 23 566 575 10.1007/s10926-013-9420-6 23358808

[B49] Schafer JL Graham JW Missing data: our view of the state of the art Psychol Meth 2002 7 147 177 12090408

