# Supplementary material for: Quality appraisal of generic self-reported instruments measuring health-related productivity changes: a systematic review
Source: BMC Public Health. 2014 Feb 4;14:115. doi: 10.1186/1471-2458-14-115 (PMC3942271; doi:10.1186/1471-2458-14-115)
Supplement: Additional file 1: Table S1 — Search strategy. [file 1471-2458-14-115-S1.pdf]

Additional file 1. Search strategy

**Medline (PubMed)**	
Construct Search	“productivity” OR “workplace productivity loss” OR “reduced workplace productivity” OR “lost workplace productivity” OR “lost productivity” OR “production capacity” OR “employability” OR “work participation” OR “work performance” OR “work functioning” OR “work limitations” OR “absenteeism” OR “sickness absence” OR “return to work” OR “sick leave” AND “presenteeism” OR "workers' compensation"
Precise validity search (measurement properties, validation studies)	(instrumentation[sh] OR Validation Studies[pt] OR ‘reproducibility of results’[MeSH Terms] OR reproducib*[tiab] OR ‘psychometrics’OR psychometr*[tiab] OR clinimetr*[tiab] OR clinometr*[tiab] OR ‘observer variation’OR observer variation[tiab] OR ‘discriminant analysis’(46)(46)(46)(46)(46)(46)(46)(46) AND reliab*[tiab] OR unreliab*[tiab] OR valid*[tiab] OR coefficient[tiab] OR homogeneity[tiab] OR homogeneous[tiab] OR ‘internal consistency’[tiab] OR (cronbach*[tiab] AND (alpha[tiab] OR alphas[tiab])) OR (item[tiab] AND (correlation*[tiab] OR selection*[tiab] OR reduction*[tiab])) OR agreement[tiab] OR precision[tiab] OR imprecision[tiab] OR ‘precise values’[tiab] OR test-retest[tiab] OR (test[tiab] AND retest[tiab]) OR (reliab*[tiab] AND (test[tiab] OR retest[tiab])) OR stability[tiab] OR interrater[tiab] OR inter-rater[tiab] OR intrarater[tiab] OR intra-rater[tiab] OR intertester[tiab] OR inter-tester[tiab] OR intratester[tiab] OR intra-tester[tiab] OR interobserver[tiab] OR inter-observer[tiab] OR intraobserver[tiab] OR intra-observer[tiab] OR intertechnician[tiab] OR inter-technician[tiab] OR intratechnician[tiab] OR intra-technician[tiab] OR interexaminer[tiab] OR inter-examiner[tiab] OR intraexaminer[tiab] OR intra-examiner[tiab] OR interassay[tiab] OR inter-assay[tiab] OR intraassay[tiab] OR intra-assay[tiab] OR interindividual[tiab] OR inter-individual[tiab] OR intraindividual[tiab] OR intra-individual[tiab] OR interparticipant[tiab] OR inter-participant[tiab] OR intraparticipant[tiab] OR intra-participant[tiab] OR kappa[tiab] OR kappa's[tiab] OR kappas[tiab] OR repeatab*[tiab] OR ((replicab*[tiab] OR repeated[tiab]) AND (measure[tiab] OR measures[tiab] OR findings[tiab] OR result[tiab] OR results[tiab] OR test[tiab] OR tests[tiab])) OR generaliza*[tiab] OR generalisa*[tiab] OR concordance[tiab] OR (intraclass[tiab] AND correlation*[tiab]) OR discriminative[tiab] OR ‘known group’[tiab] OR factor analysis[tiab] OR factor analyses[tiab] OR dimension*[tiab] OR subscale*[tiab] OR (multitrait[tiab] AND scaling[tiab] AND (analysis[tiab] OR analyses[tiab])) OR item discriminant[tiab] OR inter scale correlation*[tiab] OR error[tiab] OR errors[tiab] OR ‘individual variability’[tiab] OR (variability[tiab] AND (analysis[tiab] OR values[tiab])) OR (uncertainty[tiab] AND (measurement[tiab] OR measuring[tiab])) OR ‘standard error of measurement’[tiab] OR sensitiv*[tiab] OR responsive*[tiab] OR ((minimal[tiab] OR minimally[tiab] OR clinical[tiab] OR clinically[tiab]) AND (important[tiab] OR significant[tiab] OR detectable[tiab]) AND (change[tiab] OR difference[tiab])) OR (small*[tiab] AND (real[tiab] OR detectable[tiab]) AND (change[tiab] OR difference[tiab])) OR meaningful change[tiab] OR ‘ceiling effect’[tiab] OR ‘floor effect’[tiab] OR ‘Item response model’[tiab] OR IRT[tiab] OR Rasch[tiab] OR ‘Differential item functioning’[tiab] OR DIF[tiab] OR ‘computer adaptive testing’[tiab] OR ‘item bank’[tiab] OR ‘cross-cultural equivalence’[tiab]
Instrument search	‘questionnaires’ OR ‘questionnaire’ OR ‘self report’ OR ‘self reports’ OR ‘generic instruments’
Exclusion filter	‘addresses’[Publication Type] OR ‘biography’[Publication Type] OR ‘case reports’[Publication Type] OR ‘comment’[Publication Type] OR ‘directory’[Publication Type] OR ‘editorial’[Publication Type] OR ‘festschrift’[Publication Type] OR ‘interview’[Publication Type] OR ‘lectures’[Publication Type] OR ‘legal cases’[Publication Type] OR ‘legislation’[Publication Type] OR ‘letter’[Publication Type] OR ‘news’[Publication Type] OR ‘newspaper article’[Publication Type] OR ‘patient education handout’[Publication Type] OR ‘popular works’[Publication Type] OR ‘congresses’[Publication Type] OR ‘consensus development conference’[Publication Type] OR ‘consensus development conference, nih’[Publication Type] OR ‘practice guideline’[Publication Type]) NOT (‘animals’[MeSH Terms] NOT ‘humans’[MeSH Terms]
**Embase**	
Construct Search	‘productivity’ OR ‘workplace productivity loss’ OR ‘reduced workplace productivity’ OR ‘lost workplace productivity’ OR ‘lost productivity’ OR ‘production capacity’ OR ‘employability’ OR ‘work participation’ OR ‘work performance’ OR ‘work functioning’ OR ‘work limitations’ OR ‘absenteeism’ OR ‘sickness absence’ OR ‘return to work’ OR ‘sick leave’ AND ‘presenteeism’ OR ‘workers' compensation’
Precise validity search (measurement properties, validation studies)	'intermethod comparison'/exp OR 'data collection method'/exp OR 'validation study'/exp OR 'feasibility study'/exp OR 'pilot study'/exp OR 'psychometry'/exp OR 'reproducibility'/exp OR reproducib*:ab,ti OR 'audit':ab,ti OR psychometr*:ab,ti OR clinimetr*:ab,ti OR clinometr*:ab,ti OR 'observer variation'/exp OR 'observer variation':ab,ti OR 'discriminant analysis'/exp OR 'validity'/exp OR reliab*:ab,ti OR valid*:ab,ti OR 'coefficient':ab,ti OR 'internal consistency':ab,ti OR (cronbach*:ab,ti AND ('alpha':ab,ti OR 'alphas':ab,ti)) OR 'item correlation':ab,ti OR 'item correlations':ab,ti OR 'item selection':ab,ti OR 'item

	selections':ab,ti OR 'item reduction':ab,ti OR 'item reductions':ab,ti OR 'agreement':ab,ti OR 'precision':ab,ti OR 'imprecision':ab,ti OR 'precise values':ab,ti OR 'test-retest':ab,ti OR ('test':ab,ti AND 'retest':ab,ti) OR (reliab*:ab,ti AND ('test':ab,ti OR 'retest':ab,ti)) OR 'stability':ab,ti OR 'interrater':ab,ti OR 'inter-rater':ab,ti OR 'intrarater':ab,ti OR 'intra-rater':ab,ti OR 'intertester':ab,ti OR 'inter-tester':ab,ti OR 'intratester':ab,ti OR 'intra-tester':ab,ti OR 'interobeserver':ab,ti OR 'inter-observer':ab,ti OR 'intraobserver':ab,ti OR 'intra-observer':ab,ti OR 'intertechnician':ab,ti OR 'inter-technician':ab,ti OR 'intratechnician':ab,ti OR 'intra-technician':ab,ti OR 'interexaminer':ab,ti OR 'inter-examiner':ab,ti OR 'intraexaminer':ab,ti OR 'intra-examiner':ab,ti OR 'interassay':ab,ti OR 'inter-assay':ab,ti OR 'intraassay':ab,ti OR 'intra-assay':ab,ti OR 'interindividual':ab,ti OR 'inter-individual':ab,ti OR 'intraindividual':ab,ti OR 'intra-individual':ab,ti OR 'interparticipant':ab,ti OR 'inter-participant':ab,ti OR 'intraparticipant':ab,ti OR 'intra-participant':ab,ti OR 'kappa':ab,ti OR 'kappas':ab,ti OR 'coefficient of variation':ab,ti OR repeatab*:ab,ti OR (replicab*:ab,ti OR 'repeated':ab,ti AND ('measure':ab,ti OR 'measures':ab,ti OR 'findings':ab,ti OR 'result':ab,ti OR 'results':ab,ti OR 'test':ab,ti OR 'tests':ab,ti)) OR generaliza*:ab,ti OR generalisa*:ab,ti OR 'concordance':ab,ti OR ('intraclass':ab,ti AND correlation*:ab,ti) OR 'discriminative':ab,ti OR 'known group':ab,ti OR 'factor analysis':ab,ti OR 'factor analyses':ab,ti OR 'factor structure':ab,ti OR 'factor structures':ab,ti OR 'dimensionality':ab,ti OR subscale*:ab,ti OR 'multitrait scaling analysis':ab,ti OR 'multitrait scaling analyses':ab,ti OR 'item discriminant':ab,ti OR 'interscale correlation':ab,ti OR 'interscale correlations':ab,ti OR ('error':ab,ti OR 'errors':ab,ti AND (measure*:ab,ti OR correlat*:ab,ti OR evaluat*:ab,ti OR 'accuracy':ab,ti OR 'accurate':ab,ti OR 'precision':ab,ti OR 'mean':ab,ti)) OR 'individual variability':ab,ti OR 'interval variability':ab,ti OR 'rate variability':ab,ti OR 'variability analysis':ab,ti OR ('uncertainty':ab,ti AND ('measurement':ab,ti OR 'measuring':ab,ti)) OR 'standard error of measurement':ab,ti OR sensitiv*:ab,ti OR responsive*:ab,ti OR ('limit':ab,ti AND 'detection':ab,ti) OR 'minimal detectable concentration':ab,ti OR interpretab*:ab,ti OR (small*:ab,ti AND ('real':ab,ti OR 'detectable':ab,ti) AND ('change':ab,ti OR 'difference':ab,ti)) OR 'meaningful change':ab,ti OR 'minimal important change':ab,ti OR 'minimal important difference':ab,ti OR 'minimally important change':ab,ti OR 'minimally important difference':ab,ti OR 'minimal detectable change':ab,ti OR 'minimal detectable difference':ab,ti OR 'minimally detectable change':ab,ti OR 'minimally detectable difference':ab,ti OR 'minimal real change':ab,ti OR 'minimal real difference':ab,ti OR 'minimally real change':ab,ti OR 'minimally real difference':ab,ti OR 'ceiling effect':ab,ti OR 'floor effect':ab,ti OR 'item response model':ab,ti OR 'irt':ab,ti OR 'rasch':ab,ti OR 'differential item functioning':ab,ti OR 'dif':ab,ti OR 'computer adaptive testing':ab,ti OR 'item bank':ab,ti OR 'cross-cultural equivalence':ab,ti
Instrument search	‘questionnaires’ OR ‘questionnaire’ OR ‘self report’ OR ‘self reports’ OR ‘generic instruments’
**PsychINFO (EBSCOhost)**	
Construct Search	“productivity” OR “workplace productivity loss” OR “reduced workplace productivity” OR “lost workplace productivity” OR “lost productivity” OR “production capacity” OR “employability” OR “work participation” OR “work performance” OR “work functioning” OR “work limitations” OR “absenteeism” OR “sickness absence” OR “return to work” OR “sick leave” AND “presenteeism” OR "workers' compensation"
Precise validity search (measurement properties, validation studies)	instrumentation OR Validation Studies OR ‘reproducibility of results’ OR reproducib*OR ‘psychometrics’ OR psychometr* OR clinimetr* OR clinometr* reliab* OR unreliab* OR valid*OR ‘internal consistency’ OR item OR correlation* OR selection* OR reduction*) OR test-retest OR stability OR interrater OR inter-rater OR intrarater OR intra-rater OR intertester OR inter-tester OR intratester OR intra-tester OR interobserver OR inter-observer OR intraobserver OR intra-observer OR repeatab* replica* OR generaliza*OR generalisa*OR inter scale correlation*OR error OR errors OR ‘individual variability’ OR analysis OR values OR uncertainty measurement OR measuring OR ‘standard error of measurement’ OR sensitiv* OR ‘cross-cultural equivalence’
Instrument search	‘questionnaires’ OR ‘questionnaire’ OR ‘self report’ OR ‘self reports’ OR ‘generic instruments’
**EconLit**	
Construct Search	“productivity” OR “workplace productivity loss” OR “reduced workplace productivity” OR “lost workplace productivity” OR “lost productivity” OR “production capacity” OR “employability” OR “work participation” OR “work performance” OR “work functioning” OR “work limitations” OR “absenteeism” OR “sickness absence” OR “return to work” OR “sick leave” AND “presenteeism” OR "workers' compensation"
Precise validity search (measurement properties, validation studies)	instrumentation OR Validation Studies OR ‘reproducibility of results’ OR reproducib*OR ‘psychometrics’ OR psychometr* OR clinimetr* OR clinometr*AND reliab* OR unreliab* OR valid*OR ‘internal consistency’ OR item OR correlation* OR selection* OR reduction*) OR test-retest OR stability OR integrate OR inter-rater OR indra rater OR indra-rater OR interested OR inter-tester OR indra tester OR indra-tester OR inter Observer OR inter-observer OR indra Observer OR indra-observer OR repeatab*
Instrument search	‘questionnaires’ OR ‘questionnaire’ OR ‘self report’ OR ‘self reports’ OR ‘generic instruments’
