# Supplementary material for: Quality appraisal of generic self-reported instruments measuring health-related productivity changes: a systematic review
Source: BMC Public Health. 2014 Feb 4;14:115. doi: 10.1186/1471-2458-14-115 (PMC3942271; doi:10.1186/1471-2458-14-115)
Supplement: Additional file 2: Table S2 — Detailed description of the generic self-reported instruments measuring health-related productivity changes. [file 1471-2458-14-115-S2.pdf]

Additional file 2. Detailed description of the generic self-reported instruments measuring health-related productivity changes

Instrument	Construct / Content	# questions / items	Rating	Item example	Recall period	Discipline
Work Limitations Questionnaire (WLQ) (18, 20, 26, 27, 35, 36, 41, 43)	Degree to which health problems interfere with specific aspects of job performance and its productivity impact	16 or 25<br>4 dimensions: physical demands, time management, output demands, and mental/interpersonal demands	5-point scale form none of the time (0%) to all of the time (100%)	"In the past two weeks, how much of the time did your physical health or emotional problems make it difficult for you to stick to a routine or schedule?"	2 to 4 weeks	Medical
Endicott Work Productivity Scale (EWPS)<br>(18, 28)	Wide variety of mental and medical disorders working in a wide variety of job settings to assess the degree to which this affects the work functioning of a subject	25	5-point scale of how often the behaviour or feeling, or attitude has been manifested during the past week: 0 (best possible score) to 100 (worst possible score)	"During the past week, how frequently did you find you have forgotten to call someone?"	7 days	Medical
Work Productivity and Activity Impairment Instrument (WPAI)<br>(18, 21, 38)	The effect of general health and symptom severity on work productivity and regular activities	6	5-point scale of how severity of symptoms interfere with performance: 0 ('very severe') to 5 ('did not have symptom')	"Are you currently employed (working for pay)?"	7 days	Economics
Work Productivity Short Inventory (WPSI) (18, 20, 30)	The prevalence of medical problems that may influence work productivity and the financial implications thereof	22	Open questions	"During the past year, did you experience an anxiety disorder, such as Generalized Anxiety Disorder, Panic Disorder, Phobias, Obsessive Compulsive Disorder, or Post-traumatic Stress Disorder?"	2 weeks,<br>3 months,<br>1 year	Social sciences
American Medical Association-Guides (AMA-guide) (29)	The loss of function and determination of compensation and ability to work after injury or illness	15	10-point scale how pain now affects functioning in everyday activities: 0 ('work normally') to 10 ('unable to work at all')	"Does your pain interfere with your normal work inside and outside the home?"	Now	Medical

World Health Organization Health and work Performance Questionnaire (WHO HPQ) (31, 32)	Indirect workplace costs of illness by assessing absenteeism, presenteeism, and critical incidents	3 categories:<br>Health (31 items)<br>Work (16 items)<br>Demographics (8 items)	Open questions	"Now please think of your work experiences over the past 4 weeks (28 days). In the spaces provided below, write the number of days you spent in each of the following work situations. In the past 4 weeks (28 days), how many days did you miss an entire work day because of problems with your physical or mental health? Number of days (00-28)."	Four week period or the past 7 days	Social sciences (occupational environment al medicine)
Stanford Presenteeism Scale (SPS) (33, 40)	The impact of health problems on individual performance and productivity	6 statements reflecting cognitive, emotional and behavioural aspects	Five-point scale from 1 (if you strongly disagree with the statement) to 5 (if you strongly agree with the statement)	"Statement: 'because of my health problem, the stresses of my job were much harder to handle'."	30 days	Social sciences (occupational environment al medicine)
The PROductivity and DISease Questionnaire (PRODISQ) (23)	Productivity costs by assessing the relationship between health and productivity	7 modules<br>64 questions:<br>General information (N=4), Profession, working situation and income (N=16), Absenteeism (N=4), Compensation mechanisms (N=5), Presenteeism (N=3), Organizational level (N=17), Administrative and management costs (N=15)	Open questions<br>Multiple-choice	"On the scale below, please circle how much work you've done during your last day in your normal working hours compared to a normal working day '0' (nothing) to '10' (as much as normal)."	3 months	Economics
Health-Related Productivity Questionnaire Diary (HRPQ-D) (34)	Work productivity data within clinical trials and survey data collections: data relating to health-related labour force participation	9	Open questions<br>Multiple-choice	"Hours of work outside the home missed because of 'the health problem': record the number of hours you work you missed today because of 'the health problem'. If none, put a zero here."	Daily	Social sciences (occupational environment al medicine)
Health and Labour Questionnaire (HLQ) (19, 24)	Relationship between illness and treatment and work performance: estimate the	4 modules:<br>Absence from paid work, Reduced productivity at	4-point scale from (almost) never to (almost) always	"I did go to work but as a result of health problems. I had a problem	2 weeks	Economics

	production losses (costs) and the ability to use work as an indicator of health status in the social domain	paid work, Unpaid production, and Impediments to paid and unpaid labour		concentrating.”		
Quality & Quantity Questionnaire (Q&Q) (19)	Quantity and quality of the work performed on the last working day	2	10-point scale from 0 (practically nothing and very poor quality) to 10 (normal quantity and normal quality)	"Could you indicate how much work you actually performed today during regular hours as compared to normal on the scale below?"	Last working day	Economics
Well-Being Assessment for Productivity (WBA-P) (37)	Informative evaluation of job performance loss due to well-being related barriers	12 common barriers to productivity	Individual’s ability to perform his or her best at work (not at all, some, or a lot) Work-related and personal barriers (scoring from 0 'not at all' to 100 'a lot for all 11 reasons')	"How often have you had trouble at work concentrating or doing you best because of …? (each of the 11 items)"	4 weeks	Social sciences
Health and Work Questionnaire (HWQ) (39)	Workplace productivity and worker health	24	Multi-part questions on a 10 point-scale	"How satisfied were you overall with your job this week?"	7 days	Social sciences
Work and Health Interview (WHI) (42)	Captures data on absence time from work and related causes and on health-related reduced performance while at work and the health-related causes	Six module interview (first 3 obtain verbal informed consent, detailed data on employment status and usual work time, and data on episodes of 21 health problems; then a job visualization module, questions about health problems reported as the cause of missed work or reduced productivity, and data on demographics and annual salary)	Computer-assisted telephone interview (open questions and categorical response options (percentages))	Primes respondents to report on health-related lost productive time on days at work; asks detailed questions about usual tasks and activities performed at work and time allotted to each	2 weeks	Economics

Valuation of Lost Productivity questionnaire (VOLP) (22)	Assess labour input loss due to health (any physical, mental, or emotional problems or symptoms)	36 questions in 6 sections: employment status, job characteristics, absenteeism, work performance, unpaid work, and working environment	Open questions and multiple-choice	"Which of the following describes your current work situation? 1. Working full time as an employee 2. Working part time as an employee 3. Self-employed"	7 days	Social sciences
